# Supplementary material for: Universal geometric frustration in pyrochlores
Source: Nat Commun. 2018 Jul 5;9:2619. doi: 10.1038/s41467-018-05033-7 (PMC6033937; doi:10.1038/s41467-018-05033-7)
Supplement: Supplementary file 1 — Supplementary Information [file 41467_2018_5033_MOESM1_ESM.pdf]

Supplementary Information

Universal Geometric Frustration in Pyrochlores

Trump et al.

Supplementary Discussion

Crystallographic Details

When the *A*/*B* cation size ratio is between 1.46 and 1.80, the cations order to different sites and the pyrochlore structure is formed.[1] If the *A*/*B* ratio is less than 1.46, the cations fully mix and the fluorite structure is formed; when the *A*/*B* size ratio is above 1.80 then the perovskite structure is formed. Alternatively, if the cations are partially mixed, then the weberite structure is formed instead.[2] Even if the *A*/*B* size ratio is between 1.46 and 1.80, it is not uncommon for *A*/*B* site-mixing to occur due to spinodal decomposition.[3–6] Site-mixing, along with electronic effects, can lead to various other types of polyhedral distortions.

The most common polyhedral distortion involves the tetrahedral corners moving in or out–known as an all-in/all-out (AIAO) distortion. If AIAO ordering exists on a long-range scale, then the global symmetry drops from $Fd\overline{3}m$ to $F\overline{4}3m$, which is evidenced by the addition of 2*h* 0 0 reflections, as seen for $Pb_2Ru_2O_{6.5}$, due to a combination of lone pair effects and off-stoichiometry.[7] AIAO magnetic ordering also exists in $Nd_2Ir_2O_7$, evidenced by neutron diffraction.[8] If in-out ordering only exists over short-length scales, it is equivalent to 2-in/2-out (2I2O) ordering, which is expected to be disordered on long-length scales due to degenerate configurations.[9] Though these distortions are structurally similar the behavior which drives them is usually not. Due to the inherent geometric frustration in the pyrochlore structure 2I2O disorder is generally more common.

Both AIAO and 2I2O distortions can be modeled by placing the displaced cation on the 32*e*:(*x x x*) site instead of the 16*c*:(0 0 0) or 16*d*:$\left(\frac{1}{8}\ \frac{1}{8}\ \frac{1}{8}\right)$ Wyckoff positions. The difference between these two distortions is that 2*h* 0 0 reflections remain absent for 2I2O ordering, and the overall symmetry remains $Fd\overline{3}m$, despite the local structure deviating from cubic symmetry. Evidence for 2I2O disorder can still be

experimentally observed by diffuse scattering near 2*h* 0 0 reflections, evidenced by electron diffraction oriented in the [001] direction. This has been observed in the Nb-pyrochlores which exhibit 2I2O distortions due to charge disproportionation of Nb^{4+} into Nb^{3+} and Nb^{5+}.[10] This is not to be confused with an observation of 2*h* 0 0 reflections when pyrochlores are oriented in the [110] direction, using neutron or electron diffraction, where 2*h* 0 0 reflections can exist due to multiple scattering. Split peaks in X-ray absorption fine structure spectroscopy also provide evidence for 2I2O distortions, as for $Y_2Mo_2O_7$.[11] Alternatively, if displacement parameters describe rod-like anisotropic displacement ellipsoids (ADE) it can indicate either AIAO or 2I2O distortions, although they do not distinguish between the two.

Pancake-like ADE hint at an alternative type of disorder–tilting of the tetrahedron–rather than tetrahedral distortions. This is equivalent to the distortion in the structurally analogous β-cristobalite, a SiO_2 polymorph. In β-cristobalite, corner-sharing tetrahedra cooperatively tilt, allowing for an increase in the Si-O bond length and a deviation of the O-Si-O angle from 180°.[12] The distortion lowers the local symmetry to $P4_32_12$, resulting from tetrahedral rotations in all three directions. This may also result in only short-range ordering due to degenerate configurations. Equivalent to pancake-like ADE, cation preference for the 96*g*(*z x x*) or 96*h*(*0 y -y*) Wyckoff positions also hint at β-cristobalite distortions.

β-cristobalite distortions have already been observed for $Bi_2Ti_2O_7$ and $Bi_2Ru_2O_7$, due to lone-pair effects, as evidenced by diffuse scattering in electron diffraction,[13] pancake-like ADE, and reverse Monte-Carlo fits to pair-distribution data.[14,15] This type of distortion was also proposed for $La_2Zr_2O_7$, due to diffuse scattering in the <110> direction in electron diffraction, consistent with a β-cristobalite distortion.[16,17] A static β-cristobalite distortion is also suggested by intensity on the allowed 442 reflection due to *A*, *B*, or O displacements.[18]

Crystallographically, it is challenging to distinguish between dynamic, i.e. fluctuations around an ideal position, and static, i.e. an averaged position that is displaced from the ideal, distortions. Though

alternative Wyckoff positions, ADE, and diffuse electron scattering hint as disorder, they do not differentiate between dynamic and static displacements. Only the addition of forbidden reflections or extra intensity for allowed reflections can indicate that displacements are long-range ordered and static. Alternatively, local probes, such as pair-distribution function (PDF) analysis, NMR, X-ray absorption fine structure spectroscopy, or high-resolution transmission electron microscopy (HRTEM), can distinguish between static or dynamic distortions. However, their effectiveness is limited by the timescale and resolution of the measurements – as well as the magnitude of the displacement.

Crystallographic Tests

Fixed refinement tests were conducted on all samples for both powder diffraction and pair-distribution function (PDF) analyses using Topas Academic and GSAS-II[54]. Structures were visualized using VESTA.[55] For these systematic tests variables were fixed at small intervals, thermal parameters were fixed at reasonable isotropic values, and only minimal profile terms were allowed to refine. Resulting tests are shown in Supplementary Figures 2-41. *A*-site (16*c*) off-stoichiometry was modelled by placing the *B* cation on that Wyckoff position and vice versa for *B*-site (16*d*) off-stoichiometry, both while retaining a total occupancy of one. Site-mixing was modelled by placing equal amounts of the opposite cation on the two Wyckoff positions while retaining a total occupancy of one. O off-stoichiometry, due to cation off-stoichiometry, was modelled by excess on the O" 8*a* Wyckoff position and deficiency on the O' 8*b* position.

Alternative Wyckoff positions for the *A/B* cation sites were modelled as the 32*e* or 96*g*/96*h* Wyckoff positions for in/out or β-cristobalite (βC) distortions respectively. Though these alternative Wyckoff positions more accurately model large dynamic (fluctuations around an average position) displacements rather than static (change of average position) displacements, these tests do not differentiate very well between the two, especially for powder diffraction data. Rather these alternative Wyckoff positions hint at either large dynamic distortions or the existence of long-range disordered static

displacements. In/out displacements were modelled as (+x,+x,+x) (32e:(x,x,x)) while βC were modelled as (-2x,+x,+x) (96g:(z,x,x)). Lastly, cooperative displacements of O due to βC distortions were modelled as 96g(z,+x,+x) where z was allowed to freely refine as it is for the 48f:(z,1/8,1/8) site. Additionally O' displacements due to overall shifting of O'A_4 tetrahedra, rather than just tilting due to βC displacements, was modelled using the 32e(+x,+x,+x) site.

Not all systematic tests appear as conclusive as others. It is expected that if a specific test fails there should be a non-linear increase in refinement statistics, and it should be consistent across all refinement statistics reported. In some cases, not enough points were counted to see the non-linear trend, and when the refinement statistics disagree it is likely that the test is insensitive and the results are inconclusive. However, if a specific test succeeds a clear minimum should be observed, as is the case for several of the Supplementary Figures 2-41. Tests involving O for X-ray data clearly demonstrate that the high-Z *A*/*B* cations dominate the diffraction patterns, as most of these tests appear inconclusive. In comparison, the neutron systematic tests involving O appear much more conclusive. If not explicitly shown, all tests indicated O' (8b:(3/8,3/8,3/8)) does not prefer alternative Wyckoff positions and when a cation prefers the 96g site it also prefers the 96h site.

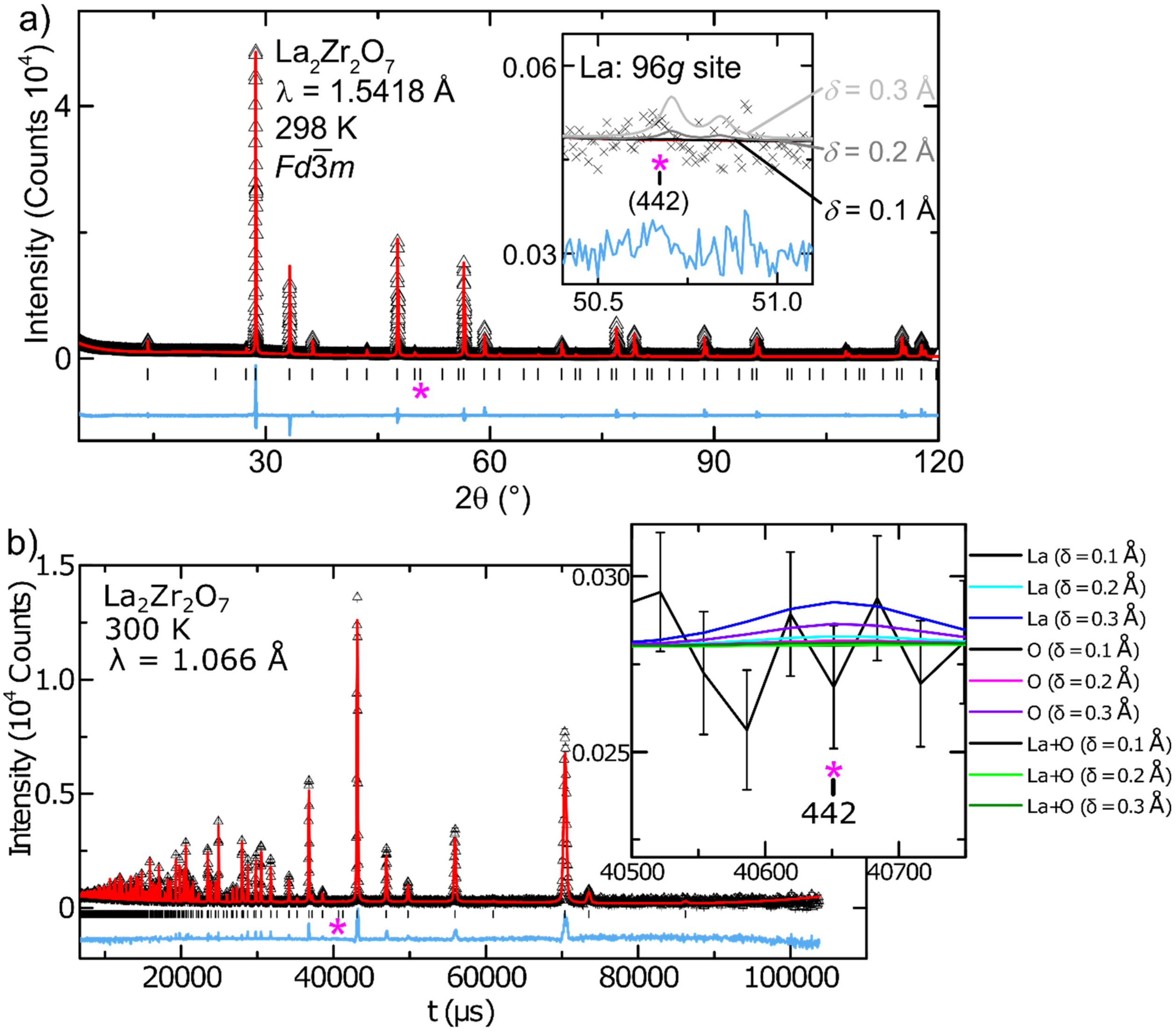

Supplementary Figure 1| Rietveld refinements on $La_2Zr_2O_7$. a) Rietveld refinement on laboratory X-ray data from a $La_2Zr_2O_7$ ground single crystal. Inset demonstrates the intensity change of the 442 reflection as a function of total La displacement when La is on the 96*g*: (*z x x*) Wyckoff position. Scatter in the difference curve in the inset is commensurate with the uncertainty. **b)** Time of flight neutron powder diffraction on a well-ground piece of the stoichiometric LZO crystal. Inset highlights the intensity of the 442 reflection as a function of total displacement δ, for La displacements, O displacements, and both La and O displacements. Data shown as black triangles, Rietveld refinement in red, and difference curve in blue for both.

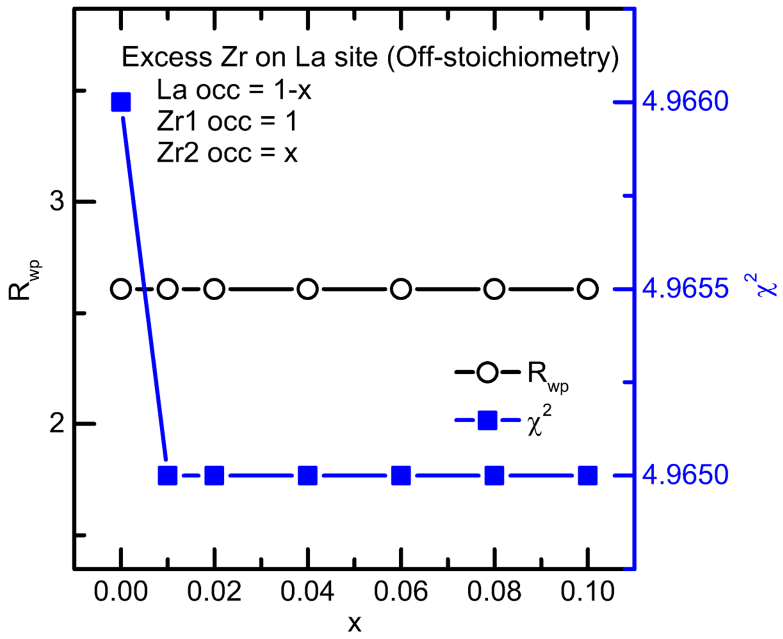

Supplementary Figure 2| Zr stuffing refinement test for LZO single crystal from time of flight neutron powder diffraction data. Here the variable x represents off-stoichiometry resulting in Zr on the La site. R_{wp} values are shown as black circles while χ2 values are shown as solid blue squares. Lines are guides to the eye. The statistics appear inconclusive.

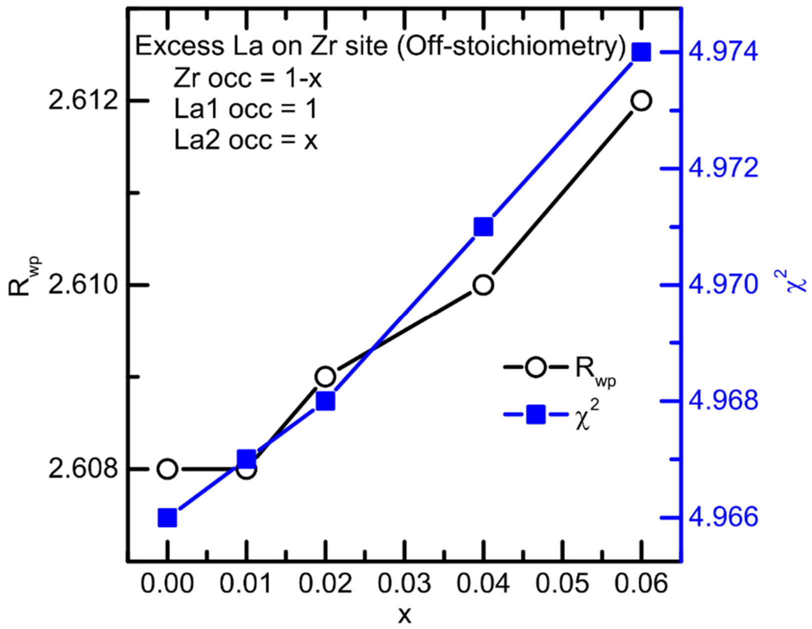

Supplementary Figure 3| La stuffing refinement test for LZO single crystal from time of flight neutron powder diffraction data. Here the variable x represents off-stoichiometry resulting in La on the Zr site. R_{wp} values are shown as black circles while χ2 values are shown as solid blue squares. Lines are guides to the eye. The statistics appear indicate a minimum for x = 0 with ideal stoichiometry.

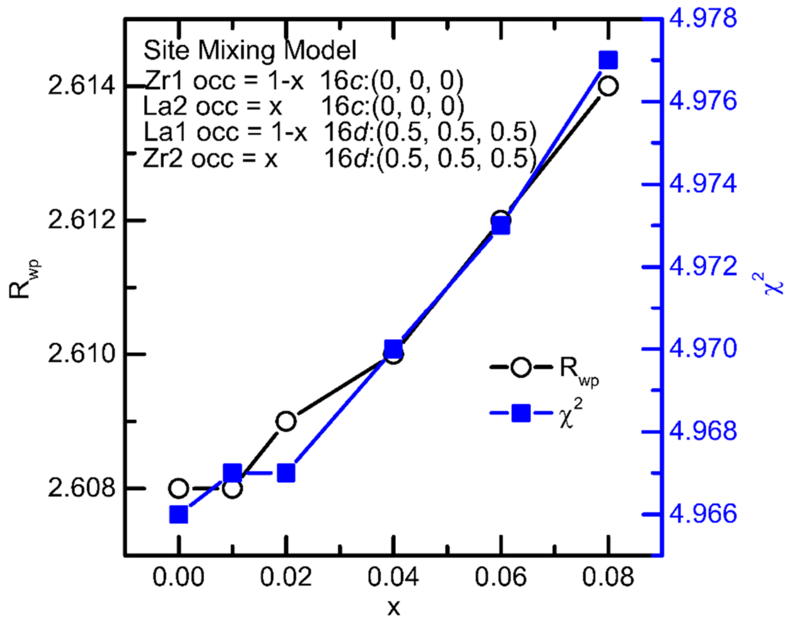

Supplementary Figure 4| Site mixing refinement test for LZO single crystal from time of flight neutron powder diffraction data. Here the variable x represents site mixing between the La and Zr sites. R_{wp} values are shown as black circles while χ2 values are shown as solid blue squares. Lines are guides to the eye. The statistics appear indicate a minimum for x = 0 with no site mixing.

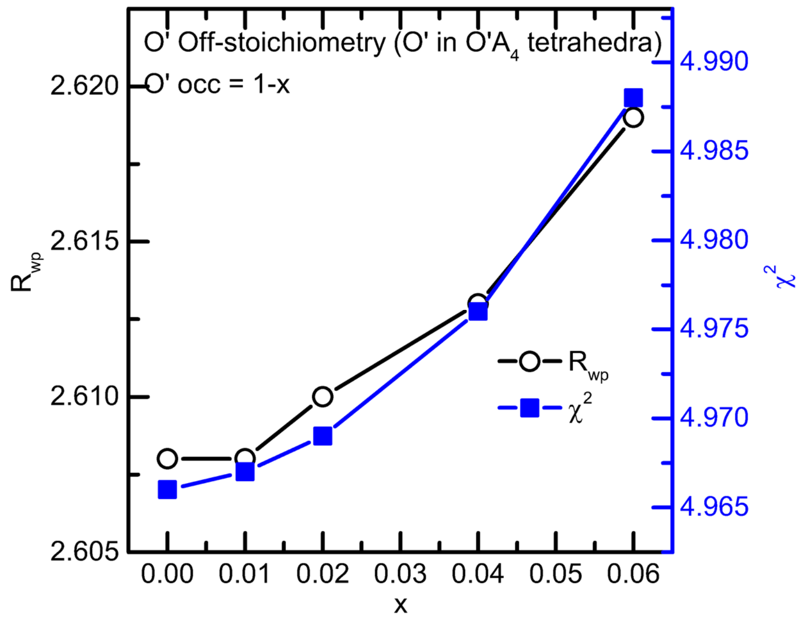

Supplementary Figure 5| O vacancy refinement test for LZO single crystal from time of flight neutron powder diffraction data. Here the variable x represents off-stoichiometry resulting in loss of O on the O' site (8*b*:(3/8,3/8,3/8)). R_{wp} values are shown as black circles while χ2 values are shown as solid blue squares. Lines are guides to the eye. The statistics appear indicate a minimum for x = 0 with ideal stoichiometry.

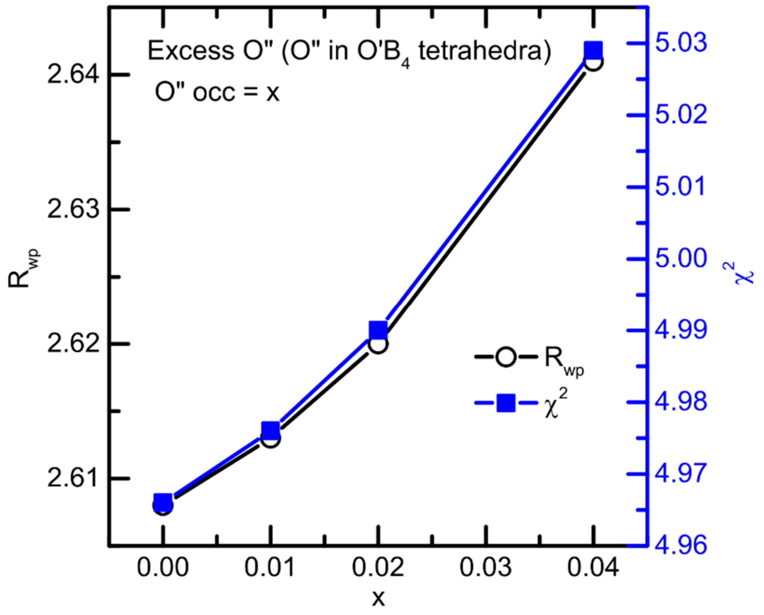

Supplementary Figure 6| Excess O refinement test for LZO single crystal from time of flight neutron powder diffraction data. Here the variable x represents off-stoichiometry resulting in O on the O” site (8*a*:(1/8,1/8,1/8)). R_{wp} values are shown as black circles while χ2 values are shown as solid blue squares. Lines are guides to the eye. The statistics appear indicate a minimum for x = 0 with ideal stoichiometry.

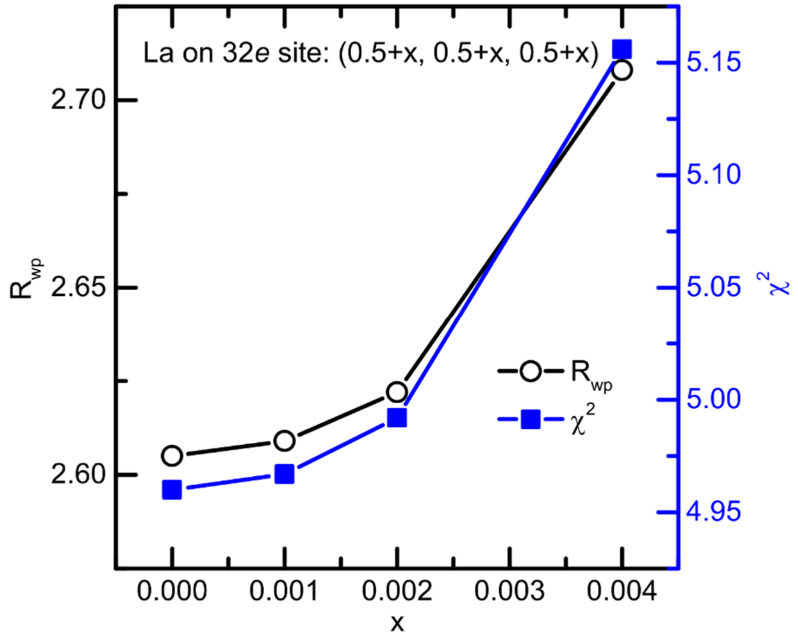

Supplementary Figure 7| Displaced La refinement test for LZO single crystal from time of flight neutron powder diffraction data. Here the variable x represents off-centering of La onto the 32*e* site. R_{wp} values are shown as black circles while χ2 values are shown as solid blue squares. Lines are guides to the eye. The statistics appear indicate a minimum for x = 0 meaning La does not prefer the 32*e* site.

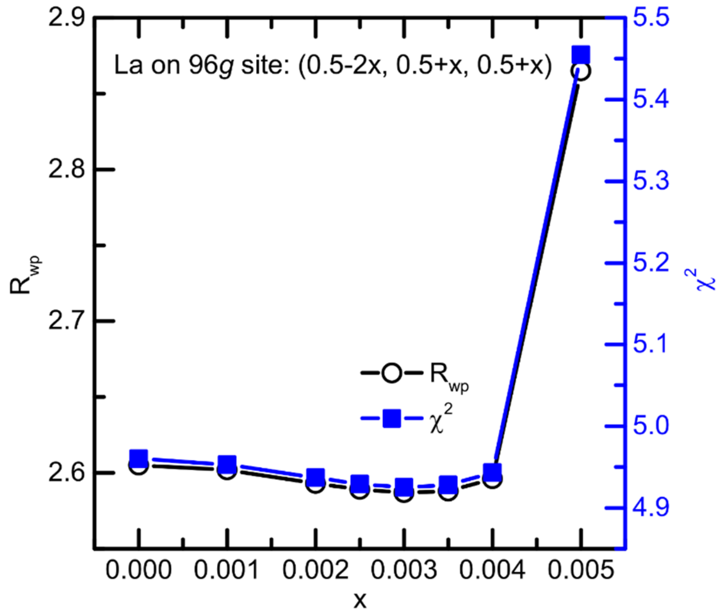

Supplementary Figure 8| Displaced La refinement test for LZO single crystal from time of flight neutron powder diffraction data. Here the variable x represents off-centering of La onto the 96*g* site. R_{wp} values are shown as black circles while χ2 values are shown as solid blue squares. Lines are guides to the eye. The statistics appear indicate a minimum for x = 0.003 (δ = 0.079 Å) meaning La does prefers to displace to the 96*g* site.

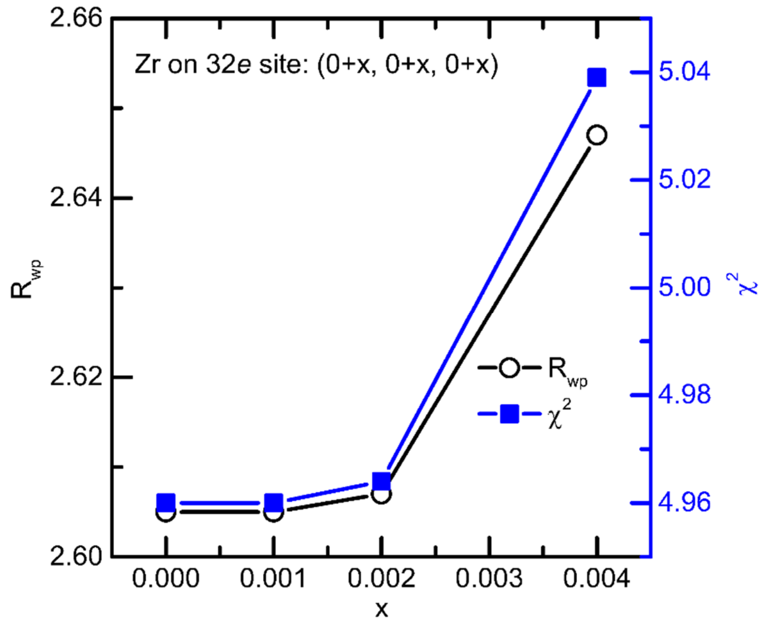

Supplementary Figure 9| Displaced Zr refinement test for LZO single crystal from time of flight neutron powder diffraction data. Here the variable x represents off-centering of Zr onto the 32*e* site. R_{wp} values are shown as black circles while χ2 values are shown as solid blue squares. Lines are guides to the eye. The statistics appear indicate a minimum for x = 0 meaning Zr does not prefer the 32*e* site.

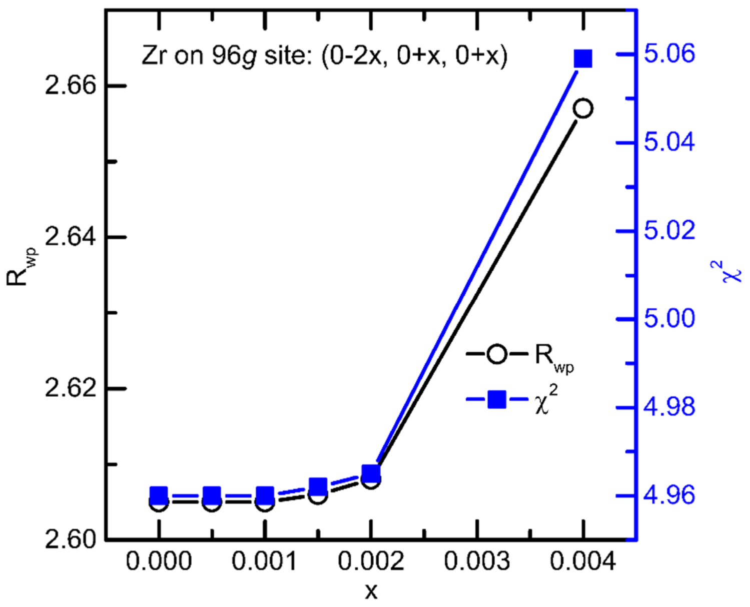

Supplementary Figure 10 | Displaced Zr refinement test for LZO single crystal from time of flight neutron powder diffraction data. Here the variable x represents off-centering of Zr onto the 96*g* site. R_{wp} values are shown as black circles while χ2 values are shown as solid squares. Lines are guides to the eye. The statistics appear indicate a minimum for x = 0-0.001, thus it is appears that Zr may not prefer the 96*g* site though a small (~0.05 Å) displacement could still be possible but not be noticeable.

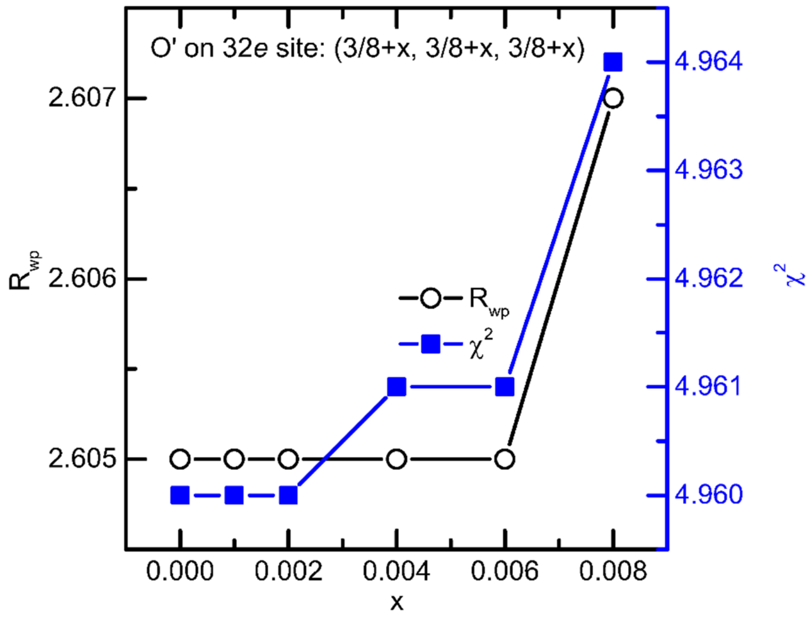

Supplementary Figure 11 | Displaced O refinement test for LZO single crystal from time of flight neutron powder diffraction data. Here the variable x represents off-centering of O' onto the 32*e* site. R_{wp} values are shown as black circles while χ2 values are shown as solid blue squares. Lines are guides to the eye. The statistics appear indicate a minimum for x = 0 meaning O prefers not to displace.

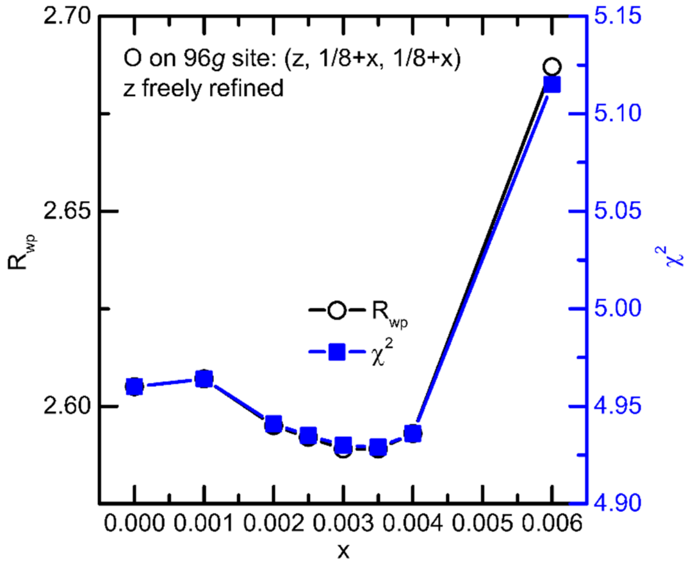

Supplementary Figure 12| Displaced O refinement test for LZO single crystal from time of flight neutron powder diffraction data. Here the variable x represents off-centering of O onto the 96*g* site. R_{wp} values are shown as black circles while χ2 values are shown as solid blue squares. Lines are guides to the eye. The statistics appear indicate a minimum for x = 0.003 (δ = 0.054 Å) meaning O does prefer to displace to the 96*g* site.

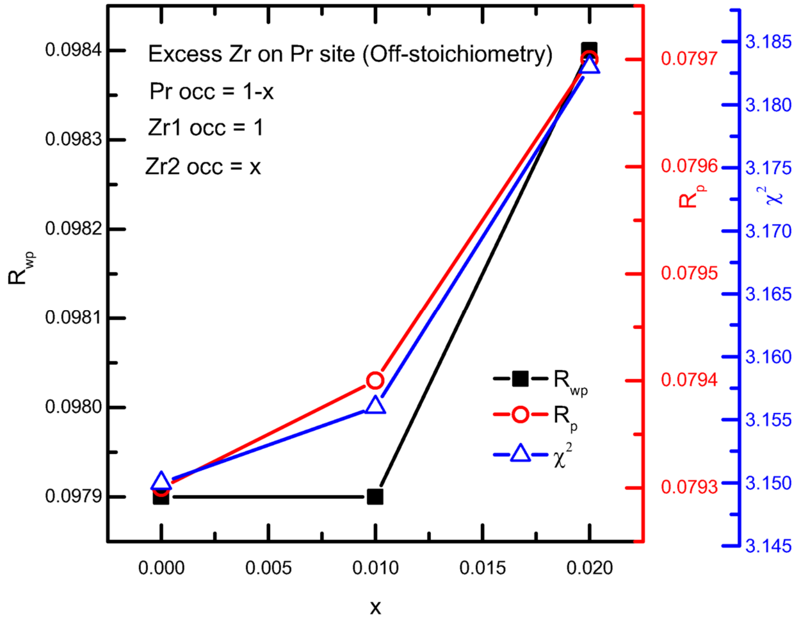

Supplementary Figure 13| Zr stuffing refinement test for PZO single crystal from synchrotron X-ray powder diffraction data. Here the variable x represents off-stoichiometry resulting in Zr on the Pr site. R_{wp} values are shown as black squares, R_p values are shown as red circles, while χ2 values are shown as blue triangles. Lines are guides to the eye. The statistics indicate a minimum at x = 0 meaning ideal stoichiometry exists.

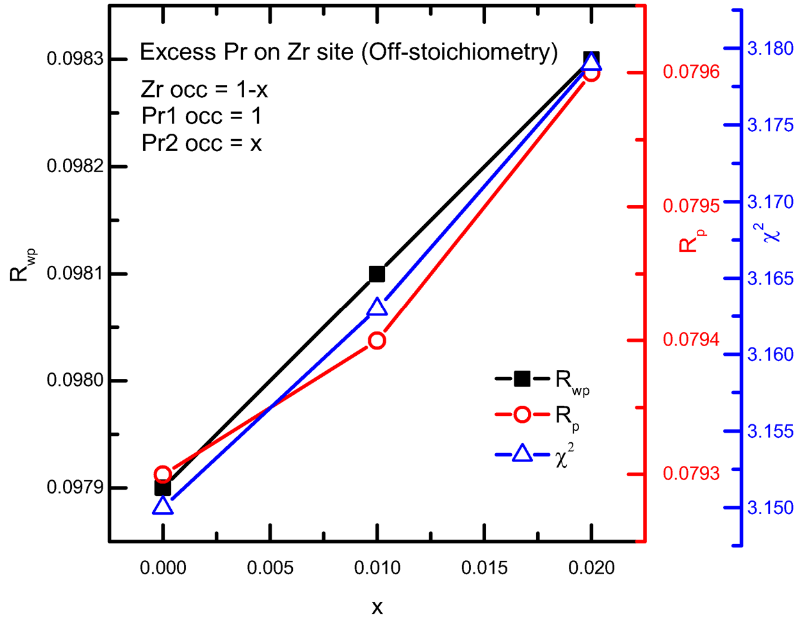

Supplementary Figure 14| Pr stuffing refinement test for PZO single crystal from synchrotron X-ray powder diffraction data. Here the variable x represents off-stoichiometry resulting in Pr on the Zr site. R_{wp} values are shown as black squares, R_p values are shown as red circles, while χ2 values are shown as blue triangles. Lines are guides to the eye. The statistics indicate a minimum at x = 0 meaning ideal stoichiometry exists.

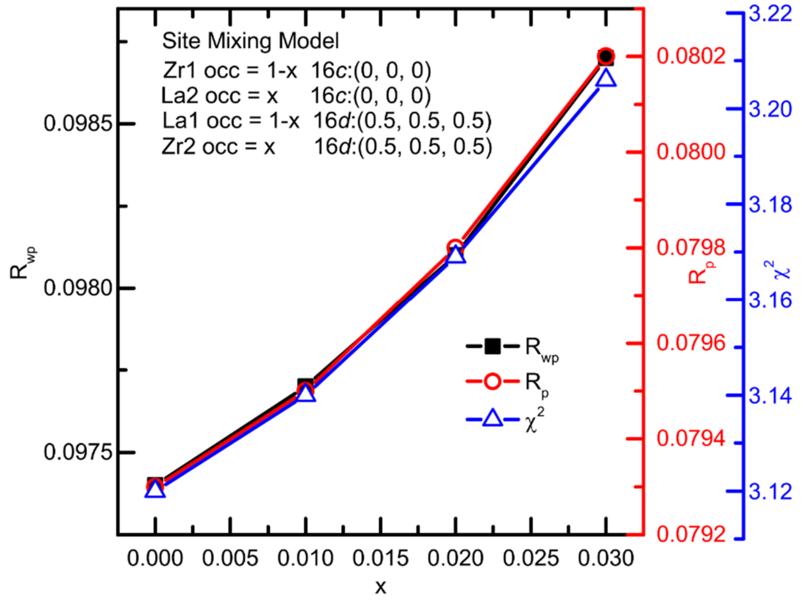

Supplementary Figure 15| Site mixing refinement test for PZO single crystal from synchrotron X-ray powder diffraction data. Here the variable x represents site mixing between the Pr and Zr sites. R_{wp} values are shown as black squares, R_p values are shown as red circles, while χ2 values are shown as blue triangles. Lines are guides to the eye. The statistics indicate a minimum at x = 0 meaning ideal site mixing exists.

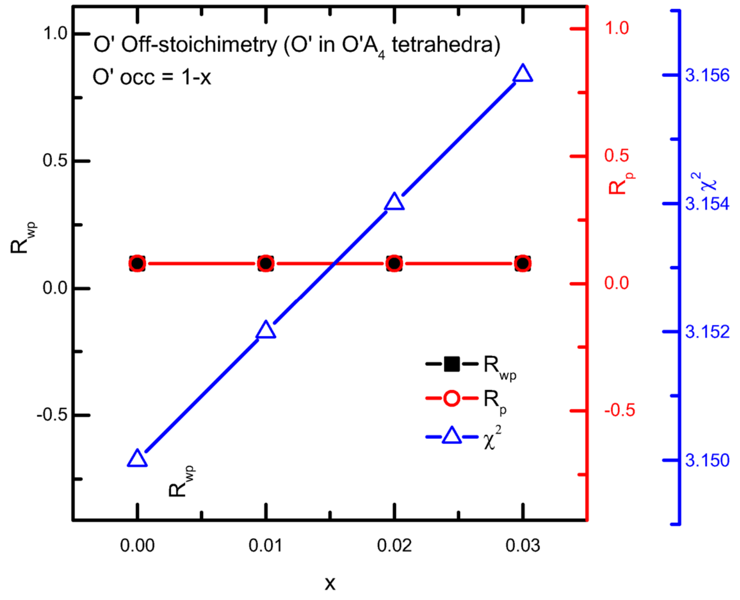

Supplementary Figure 16| O vacancy refinement test for PZO single crystal from synchrotron X-ray powder diffraction data. Here the variable x represents O vacancies on the O' site (8*b*:(3/8,3/8,3/8)). R_{wp} values are shown as black squares, R_p values are shown as red circles, while χ2 values are shown as blue triangles. Lines are guides to the eye. The statistics indicate a minimum at x = 0 meaning ideal stoichiometry exists, though statistic appear inconclusive.

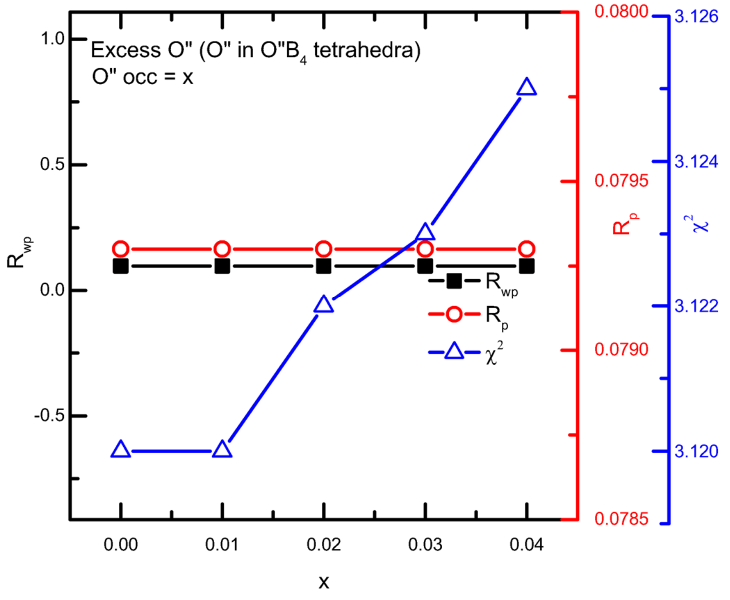

Supplementary Figure 17| Excess O refinement test for PZO single crystal from synchrotron X-ray powder diffraction data. Here the variable x represents excess O on the O" site (8*a*:(1/8,1/8,1/8)). R_{wp} values are shown as black squares, R_p values are shown as red circles, while χ2 values are shown as blue triangles. Lines are guides to the eye. The statistics indicate a minimum at x = 0 meaning ideal stoichiometry exists, though the statistics appear inconclusive.

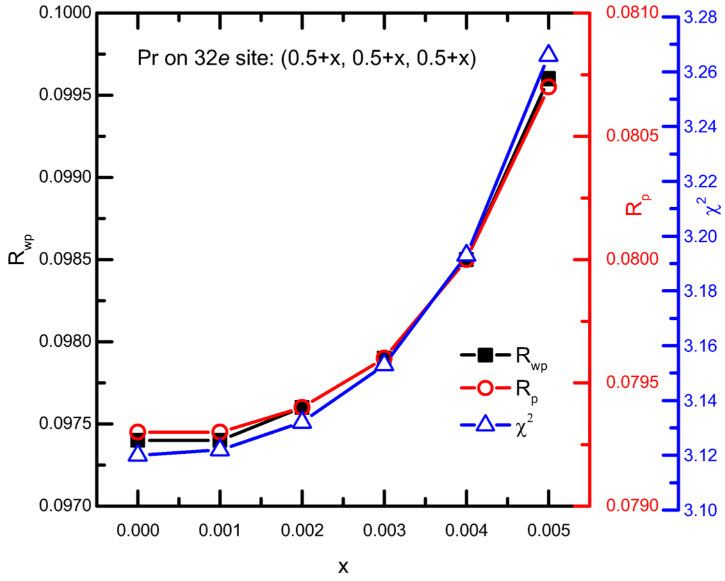

Supplementary Figure 18| Displaced Pr refinement test for PZO single crystal from synchrotron X-ray powder diffraction data. Here the variable x represents off-centering of Pr towards the 32*e* site. R_{wp} values are shown as black squares, R_p values are shown as red circles, while χ2 values are shown as blue triangles. Lines are guides to the eye. The statistics indicate a minimum at x = 0 meaning Pr does not prefer the 32e Wyckoff position.

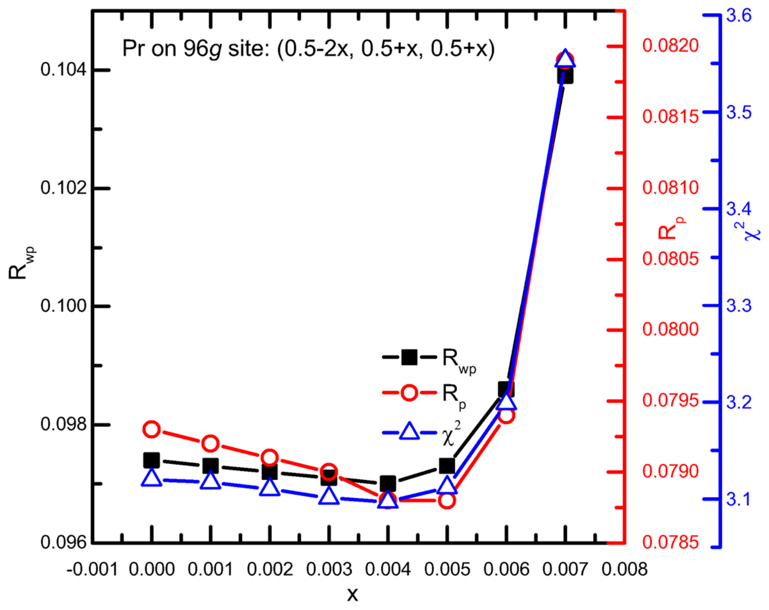

Supplementary Figure 19| Displaced refinement test for PZO single crystal from synchrotron X-ray powder diffraction data. Here the variable x represents off-centering of Pr towards the 96*g* site. R_{wp} values are shown as black squares, R_p values are shown as red circles, while χ2 values are shown as blue triangles. Lines are guides to the eye. The statistics indicate a minimum at x = 0.004 meaning Pr prefers the 96*g* Wyckoff position.

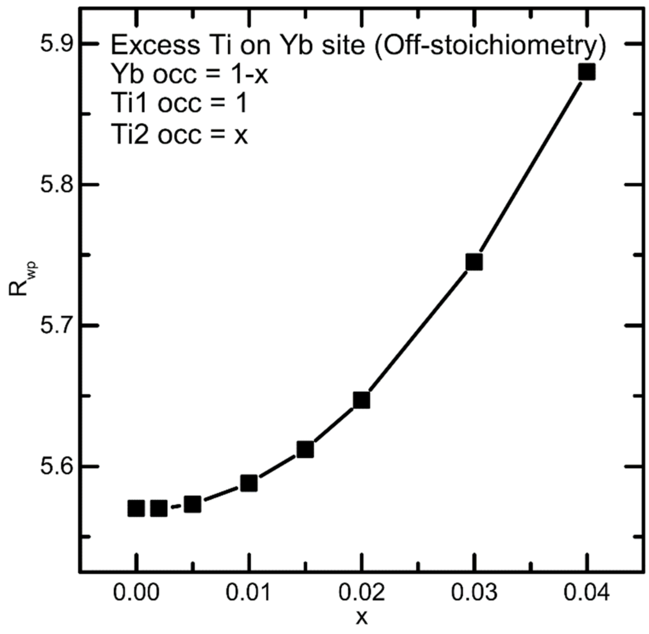

Supplementary Figure 20| Ti stuffing refinement test for YTO single crystal from laboratory X-ray powder diffraction data. Here the variable x represents off-stoichiometry resulting in Ti on the Yb site. R_{wp} values are shown as black squares. Lines are guides to the eye. The statistics indicate a minimum at x = 0 meaning ideal stoichiometry exists.

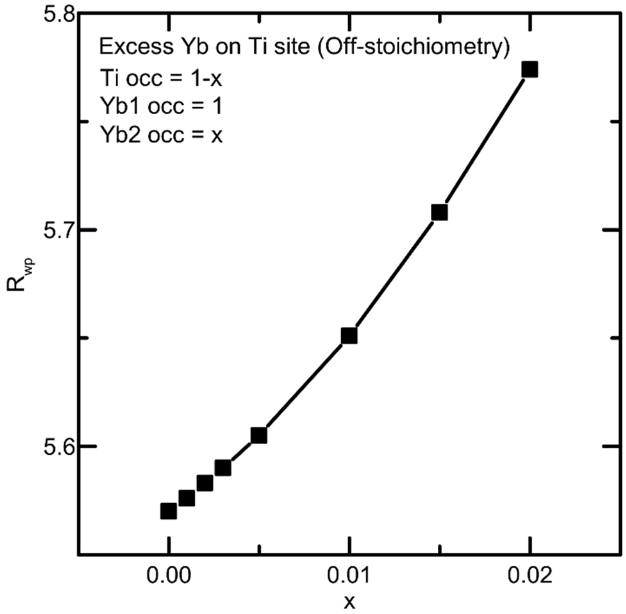

Supplementary Figure 21| Yb stuffing refinement test for YTO single crystal from laboratory X-ray powder diffraction data. Here the variable x represents off-stoichiometry resulting in Yb on the Ti site. R_{wp} values are shown as black squares. Lines are guides to the eye. The statistics indicate a minimum at x = 0 meaning ideal stoichiometry exists.

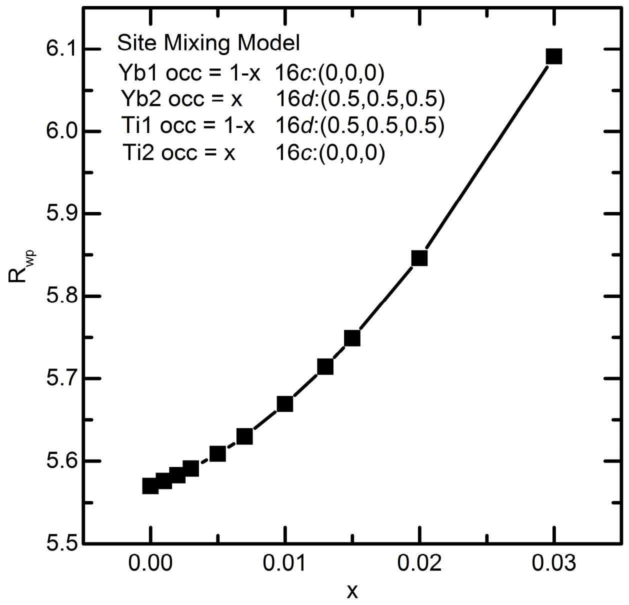

Supplementary Figure 22| Site mixing refinement test for YTO single crystal from laboratory X-ray powder diffraction data. Here the variable x represents site-mixing between the Ti and Yb sites. R_{wp} values are shown as black squares. Lines are guides to the eye. The statistics indicate a minimum at x = 0 meaning ideal site mixing occurs.

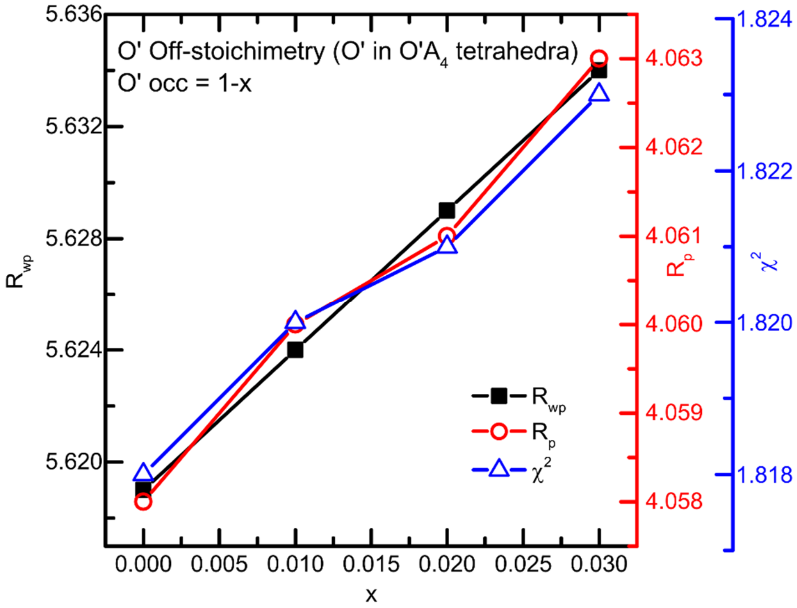

Supplementary Figure 23| O vacancy refinement test for YTO single crystal from laboratory X-ray powder diffraction data. Here the variable x represents off-stoichiometry resulting on loss of O in the O' site (8*b*:(3/8,3/8,3/8)). R_{wp} values are shown as black squares while χ2 values are shown as solid blue squares. Lines are guides to the eye. The statistics indicate a minimum at x = 0 meaning ideal stoichiometry exists.

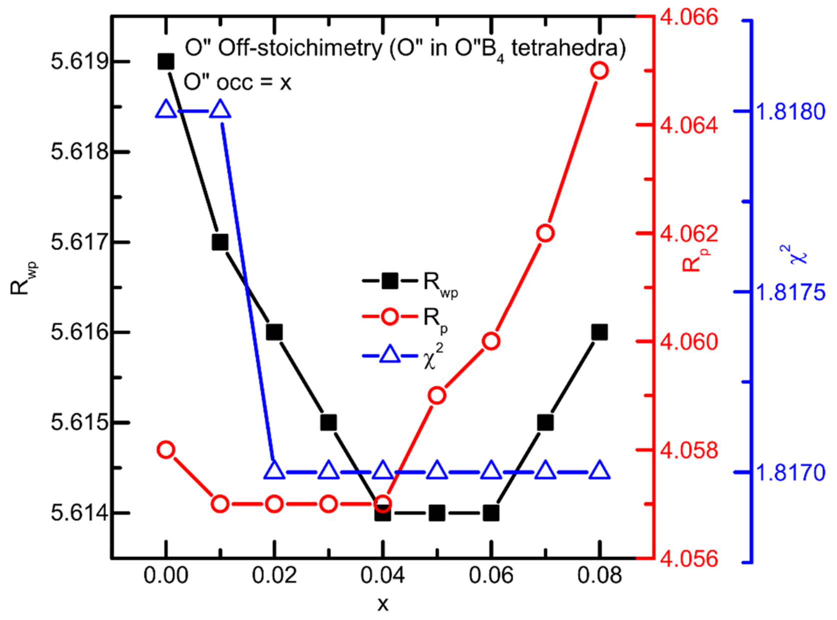

Supplementary Figure 24| Excess O refinement test for YTO single crystal from laboratory X-ray powder diffraction data. Here the variable x represents off-stoichiometry resulting in excess O in the O” site (8*a*:(1/8,1/8,1/8)). R_{wp} values are shown as black squares while χ2 values are shown as solid blue squares. Lines are guides to the eye. The statistics appear odd, possibly indicating off-stoichiometry or lack of sensitivity to O.

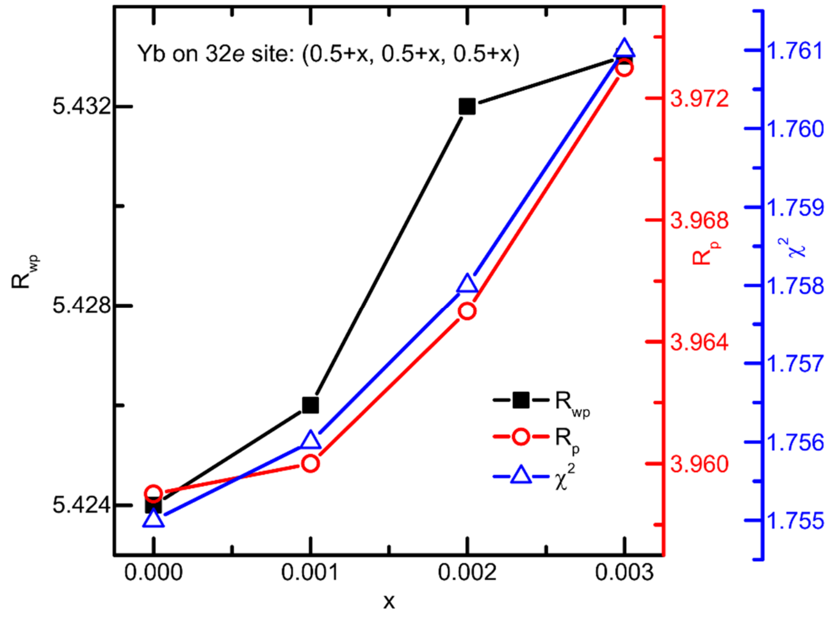

Supplementary Figure 25| Displaced Yb refinement test for YTO single crystal from laboratory X-ray powder diffraction data. Here the variable x represents off-centering of Yb to the 32*e* site. R_{wp} values are shown as black squares while χ2 values are shown as solid blue squares. Lines are guides to the eye. The statistics indicate a minimum at x = 0 indicating Yb does not prefer the 32*e* Wyckoff position.

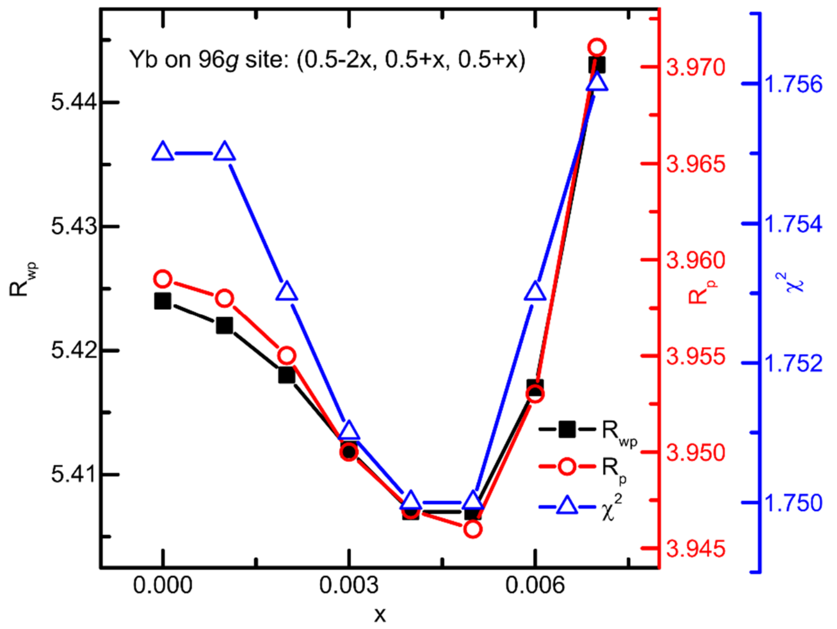

Supplementary Figure 26| Displaced Yb refinement test for YTO single crystal from laboratory X-ray powder diffraction data. Here the variable x represents off-centering of Yb to the 96*g* site. R_{wp} values are shown as black squares while χ2 values are shown as solid blue squares. Lines are guides to the eye. The statistics indicate a minimum at x = 0.005 (δ = 0.12 Å) indicating Yb prefers the 96*g* Wyckoff position.

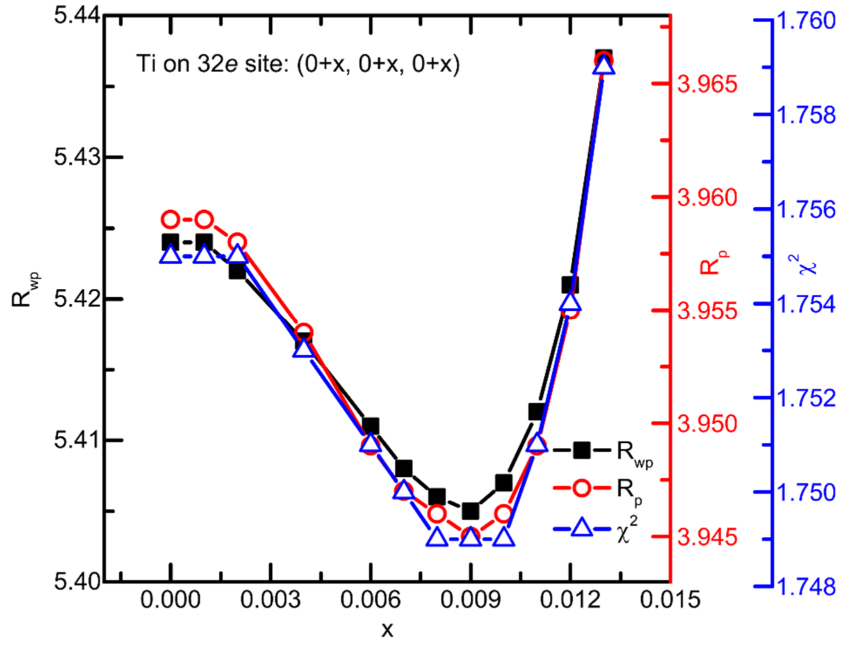

Supplementary Figure 27| Displaced Ti refinement test for YTO single crystal from laboratory X-ray powder diffraction data. Here the variable x represents off-centering of Ti to the 32*e* site. R_{wp} values are shown as black squares while χ2 values are shown as solid blue squares. Lines are guides to the eye. The statistics indicate a minimum at x = 0.009 indicating Ti prefers the 32*e* Wyckoff position.

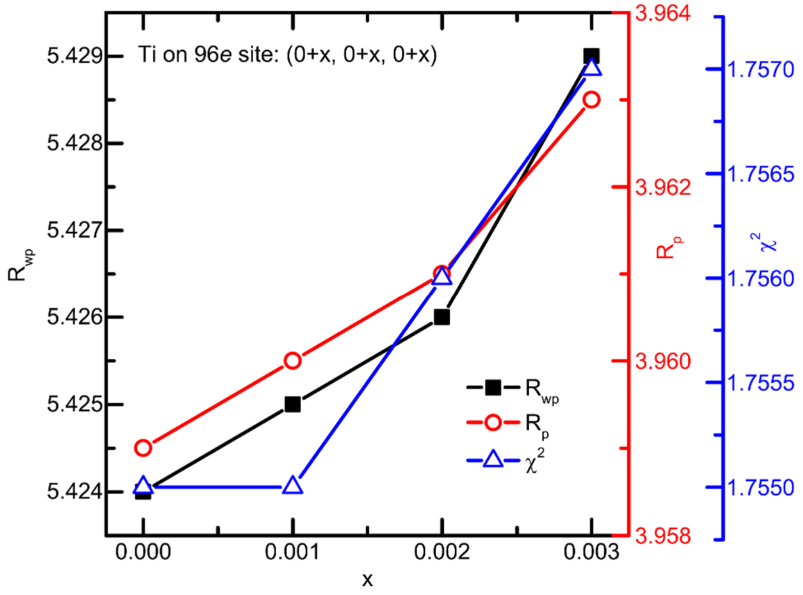

Supplementary Figure 28| Displaced Ti refinement test for YTO single crystal from laboratory X-ray powder diffraction data. Here the variable x represents off-centering of Ti to the 96*g* site. R_{wp} values are shown as black squares while χ2 values are shown as solid blue squares. Lines are guides to the eye. The statistics indicate a minimum at x = 0 indicating Ti does not prefer the 96*g* Wyckoff position.

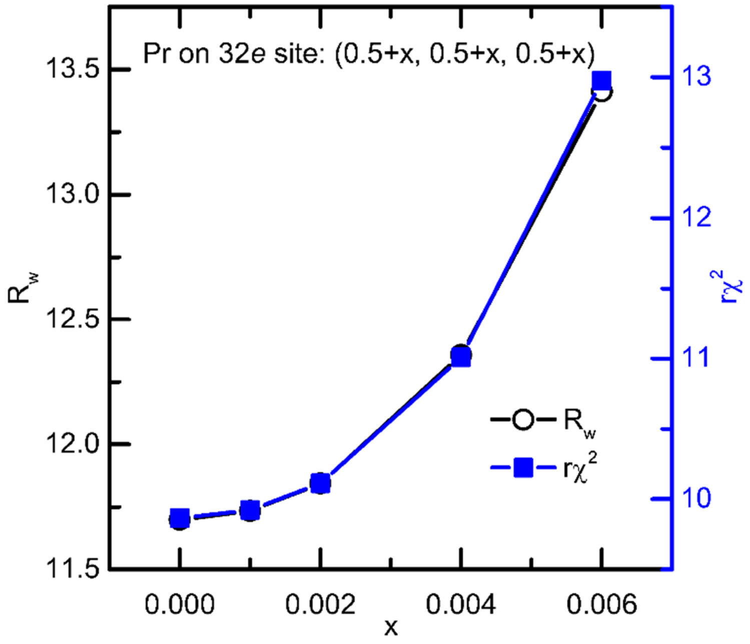

Supplementary Figure 29| Displaced Pr refinement test for sintered stoichiometric PZO sample from time of flight neutron pair-distribution function data. Here the variable x represents off-centering of Pr to the 32*e* site. R_{wp} values are shown as black circles while χ2 values are shown as solid blue squares. Lines are guides to the eye. The statistics indicate a minimum at x = 0 meaning Pr does not prefer the 32*e* Wyckoff position.

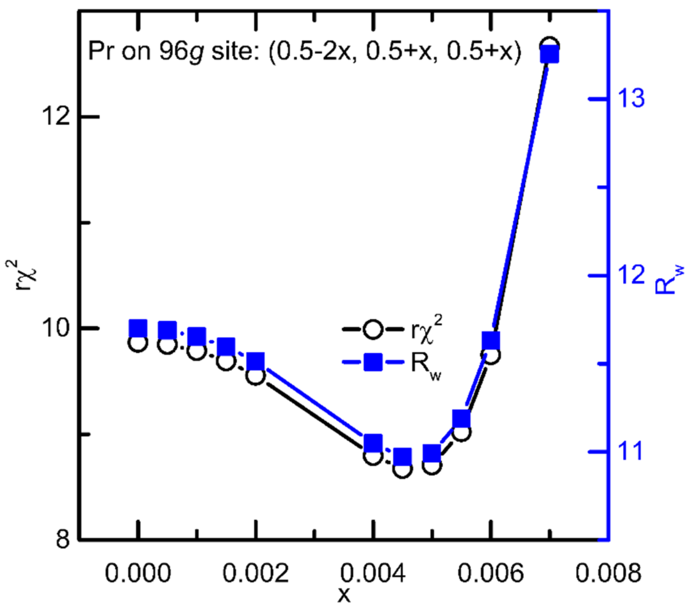

Supplementary Figure 30| Displaced Pr refinement test for sintered stoichiometric PZO sample from time of flight neutron pair-distribution function data. Here the variable x represents off-centering of Pr to the 96*g* site. R_{wp} values are shown as black circles while χ2 values are shown as solid blue squares. Lines are guides to the eye. The statistics indicate a minimum at x = 0.0045 (δ = 0.13 Å) meaning Pr prefers the 96*g* Wyckoff position.

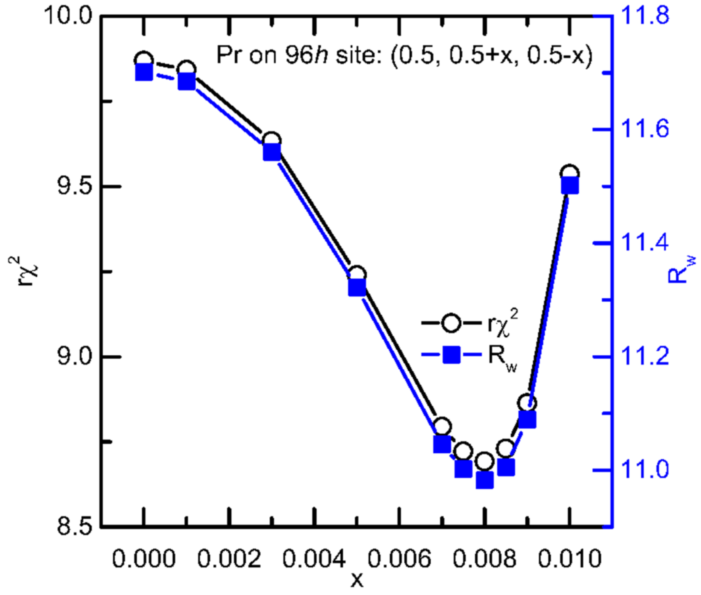

Supplementary Figure 31| Displaced Pr refinement test for sintered stoichiometric PZO sample from time of flight neutron pair-distribution function data. Here the variable x represents off-centering of Pr to the 96*h* site. R_{wp} values are shown as black circles while χ2 values are shown as solid blue squares. Lines are guides to the eye. The statistics indicate a minimum at x = 0.008 (δ = 0.12 Å) meaning Pr prefers the 96*h* Wyckoff position.

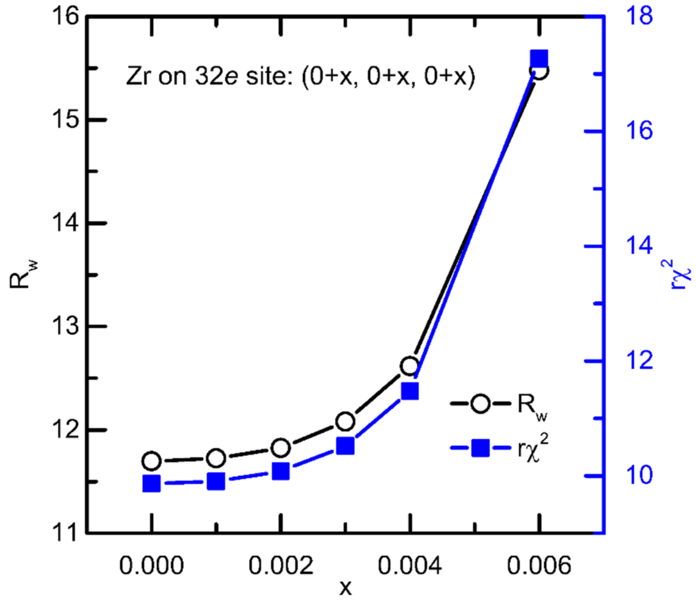

Supplementary Figure 32| Displaced Zr refinement test for sintered stoichiometric PZO sample from time of flight neutron pair-distribution function data. Here the variable x represents off-centering of Zr to the 32*e* site. R_{wp} values are shown as black circles while χ2 values are shown as solid blue squares. Lines are guides to the eye. The statistics indicate a minimum at x = 0 meaning Zr does not prefer the 32*e* Wyckoff position.

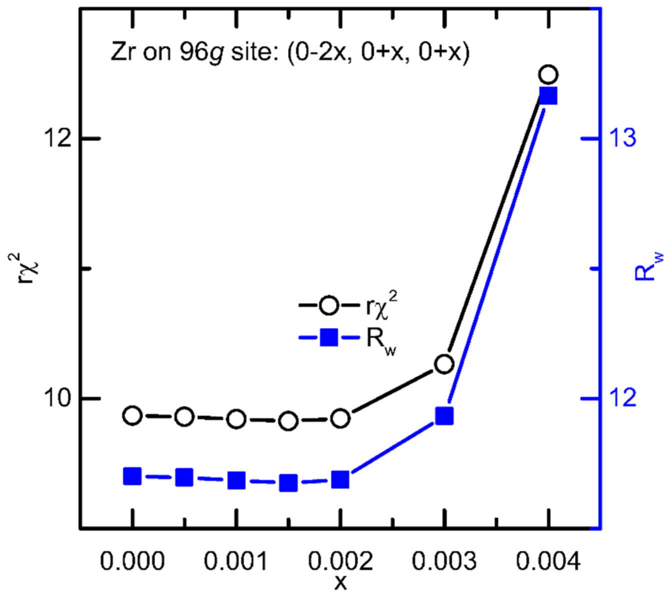

Supplementary Figure 33| Displaced Zr refinement test for sintered stoichiometric PZO sample from time of flight neutron pair-distribution function data. Here the variable x represents off-centering of Zr to the 96*g* site. R_{wp} values are shown as black circles while χ2 values are shown as solid blue squares. Lines are guides to the eye. The statistics indicate a minimum at x = 0.0015 (δ = 0.039 Å) meaning Zr prefers the 96*g* Wyckoff position.

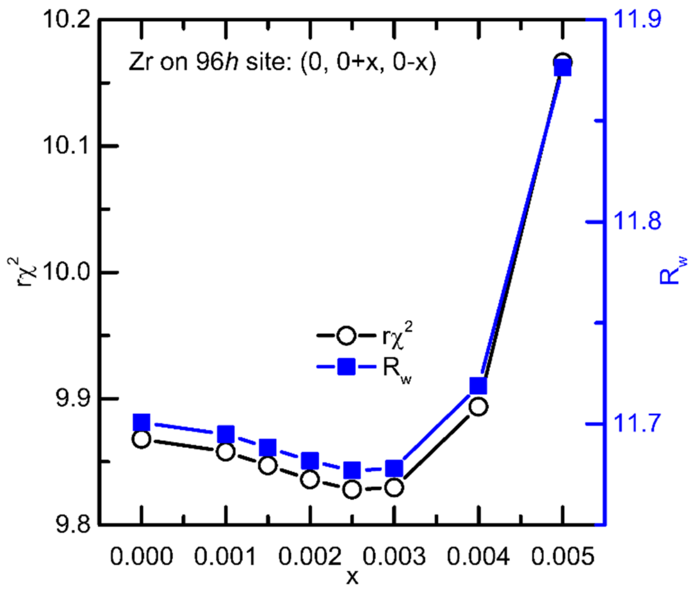

Supplementary Figure 34| Displaced Zr refinement test for sintered stoichiometric PZO sample from time of flight neutron pair-distribution function data. Here the variable x represents off-centering of Zr to the 96*h* site. R_{wp} values are shown as black circles while χ2 values are shown as solid blue squares. Lines are guides to the eye. The statistics indicate a minimum at x = 0.0025 (δ = 0.038 Å) meaning Zr prefers the 96*h* Wyckoff position.

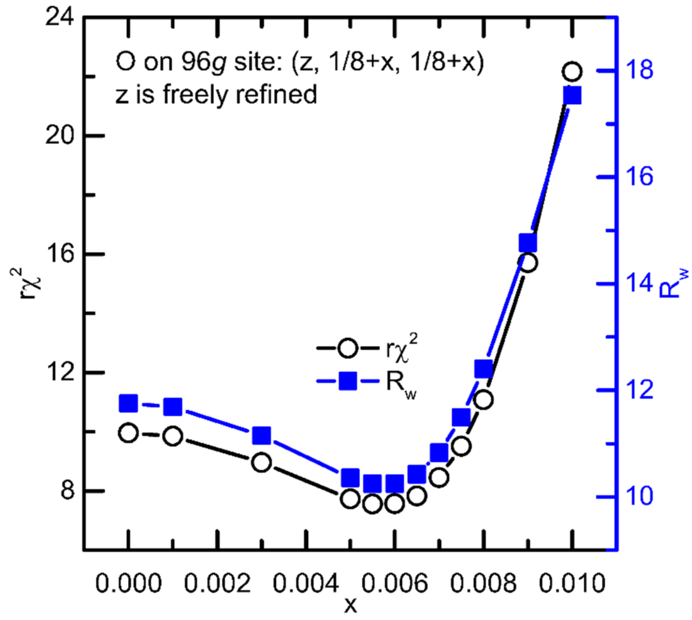

Supplementary Figure 35| Displaced O refinement test for sintered stoichiometric PZO sample from time of flight neutron pair-distribution function data. Here the variable x represents off-centering of O to the 96*g* site. R_{wp} values are shown as black circles while χ2 values are shown as solid blue squares. Lines are guides to the eye. The statistics indicate a minimum at x = 0.0055 (δ = 0.098 Å) meaning Zr prefers the 96*g* Wyckoff position.

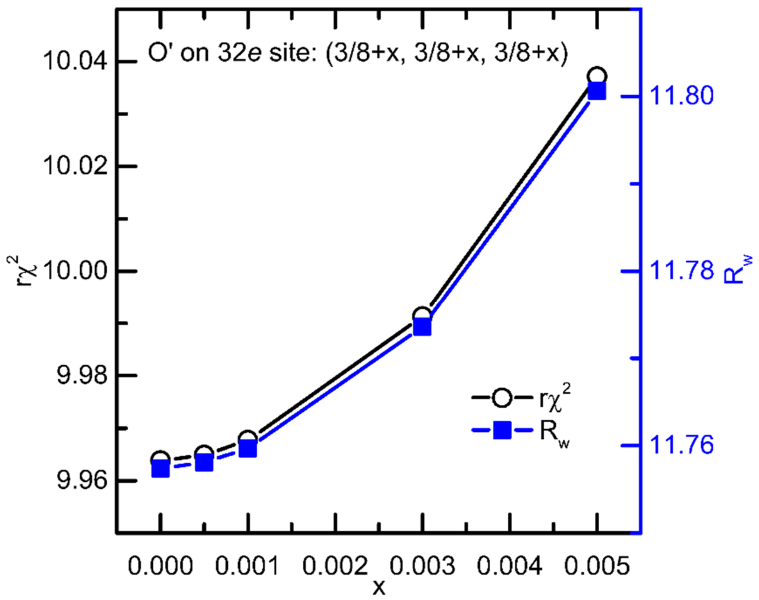

Supplementary Figure 36| Displaced refinement test for sintered stoichiometric PZO sample from time of flight neutron pair-distribution function data. Here the variable x represents off-centering of O' to the 32*e* site. R_{wp} values are shown as black circles while χ2 values are shown as solid blue squares. Lines are guides to the eye. The statistics indicate a minimum at x = 0 meaning O' does not move off-center.

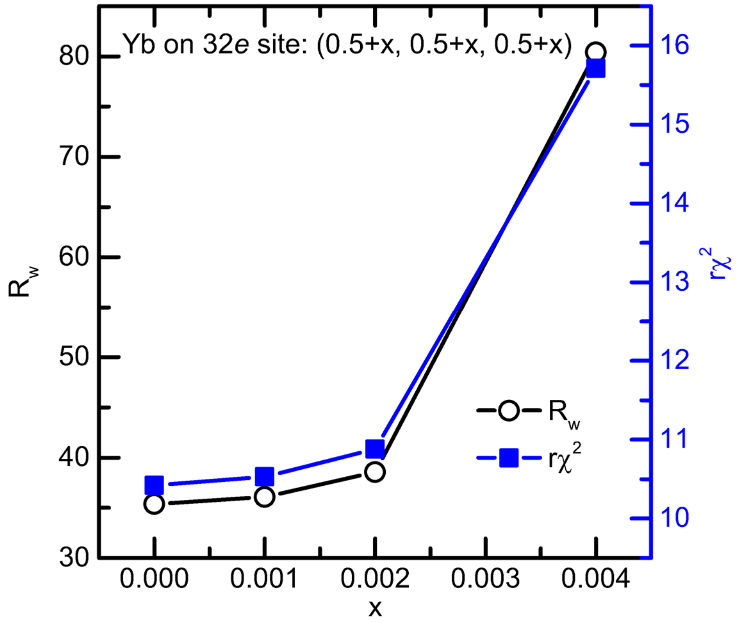

Supplementary Figure 37| Displaced Yb refinement test for YTO single crystal well ground for synchrotron X-ray pair-distribution function data. Here the variable x represents off-centering of Yb to the 32*e* site. R_{wp} values are shown as black circles while χ2 values are shown as solid blue squares. Lines are guides to the eye. The statistics indicate a minimum at x = 0 meaning Yb does not prefer the 32*e* Wyckoff position.

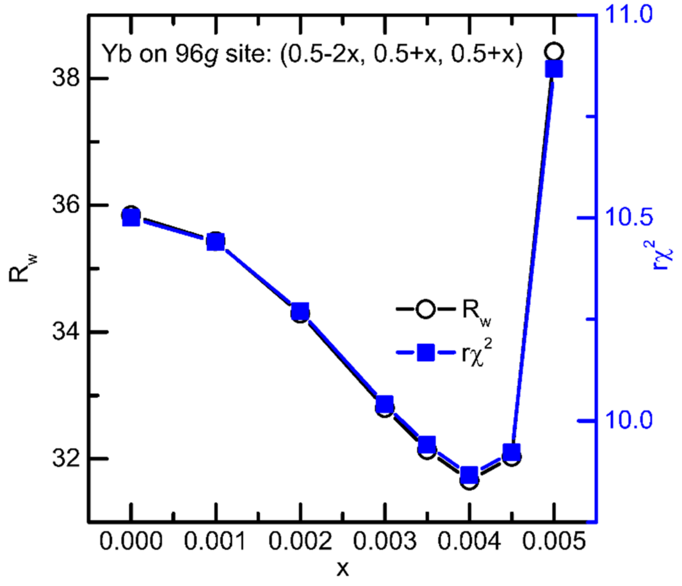

Supplementary Figure 38| Displaced Yb refinement test for YTO single crystal well ground for synchrotron X-ray pair-distribution function data. Here the variable x represents off-centering of Yb to the 96*g* site. R_{wp} values are shown as black circles while χ2 values are shown as solid blue squares. Lines are guides to the eye. The statistics indicate a minimum at x = 0.004 (δ = 0.098 Å) meaning Yb prefers the 96*g* Wyckoff position.

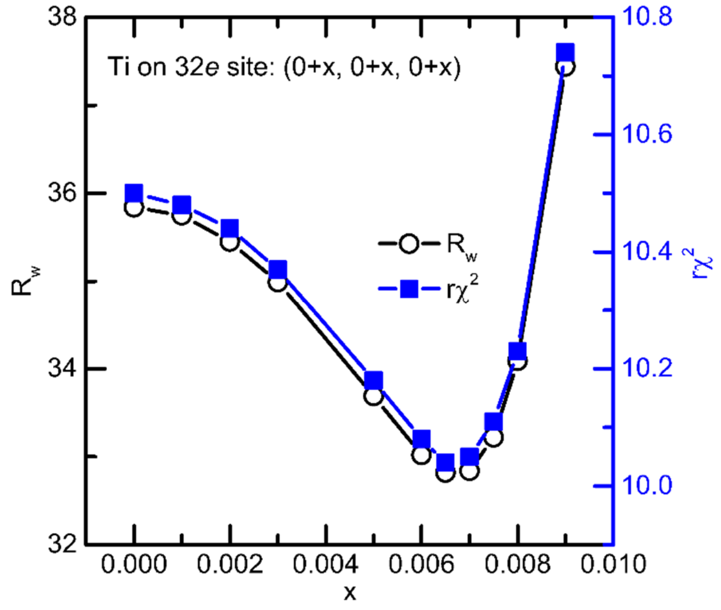

Supplementary Figure 39| Displaced refinement test for YTO single crystal well ground for synchrotron X-ray pair-distribution function data. Here the variable x represents off-centering of Ti to the 32*e* site. R_{wp} values are shown as black circles while χ2 values are shown as solid blue squares. Lines are guides to the eye. The statistics indicate a minimum at x = 0.0065 (δ = 0.11 Å) meaning Ti prefers the 32*e* Wyckoff position.

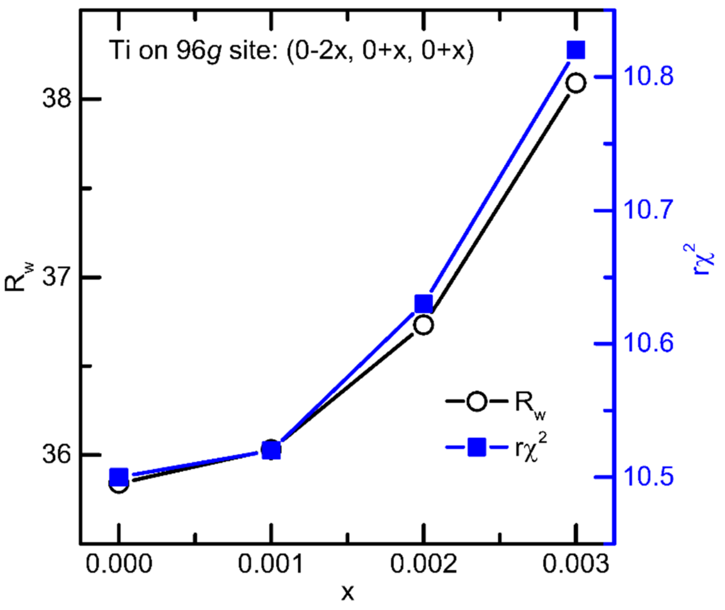

Supplementary Figure 40| Displaced Ti refinement test for YTO single crystal well ground for synchrotron X-ray pair-distribution function data. Here the variable x represents off-centering of Ti to the 96*g* site. R_{wp} values are shown as black circles while χ2 values are shown as solid blue squares. Lines are guides to the eye. The statistics indicate a minimum at x = 0 meaning Ti does not prefer the 96*g* Wyckoff position.

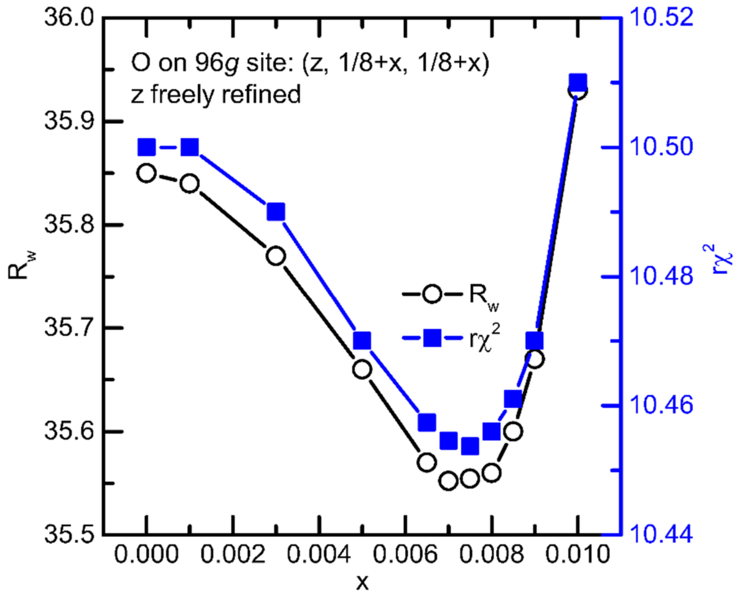

Supplementary Figure 41| Displaced O refinement test for YTO single crystal well ground for synchrotron X-ray pair-distribution function data. Here the variable x represents off-centering of O to the 96*g* site. R_{wp} values are shown as black circles while χ2 values are shown as solid blue squares. Lines are guides to the eye. The statistics indicate a minimum at x = 0.007-0.0075 meaning O prefers the 96*g* Wyckoff position.

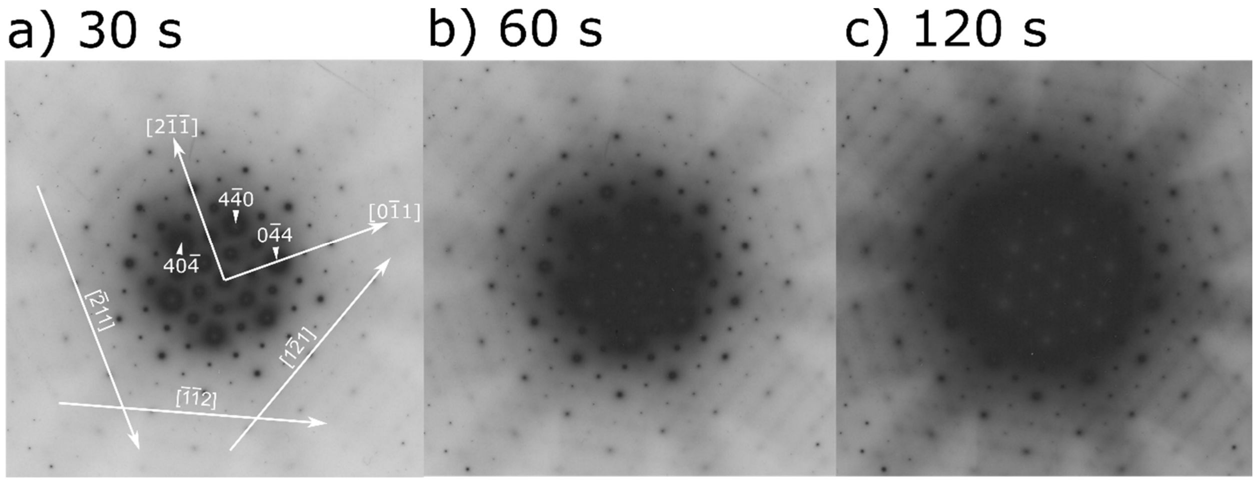

Supplementary Figure 42| Selected Area Electron Diffraction on $Pr_2Zr_2O_7$ crystal in the [111] direction. Exposure times were a) 30 s, b) 60 s, c) 120 s, d) 240 s, and e) 300 s, on film. All film was developed under similar conditions.

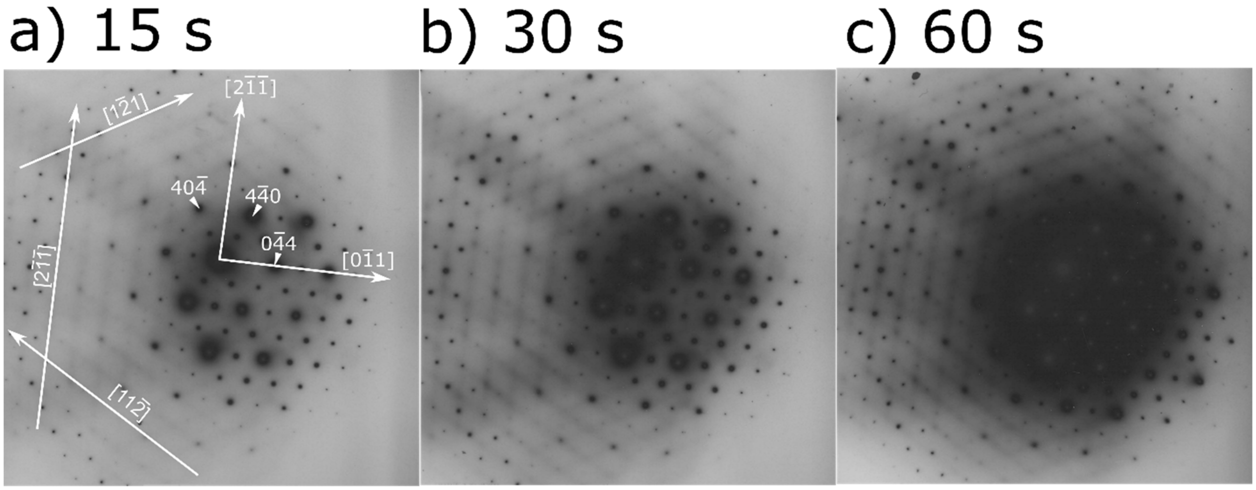

Supplementary Figure 43| Selected Area Electron Diffraction on $Yb_2Ti_2O_7$ crystal in the [111] direction. Exposure times were a) 15 s, b) 30 s, c) 60 s, d) 120 s, and e) 240 s, on film. All film was developed under similar conditions.

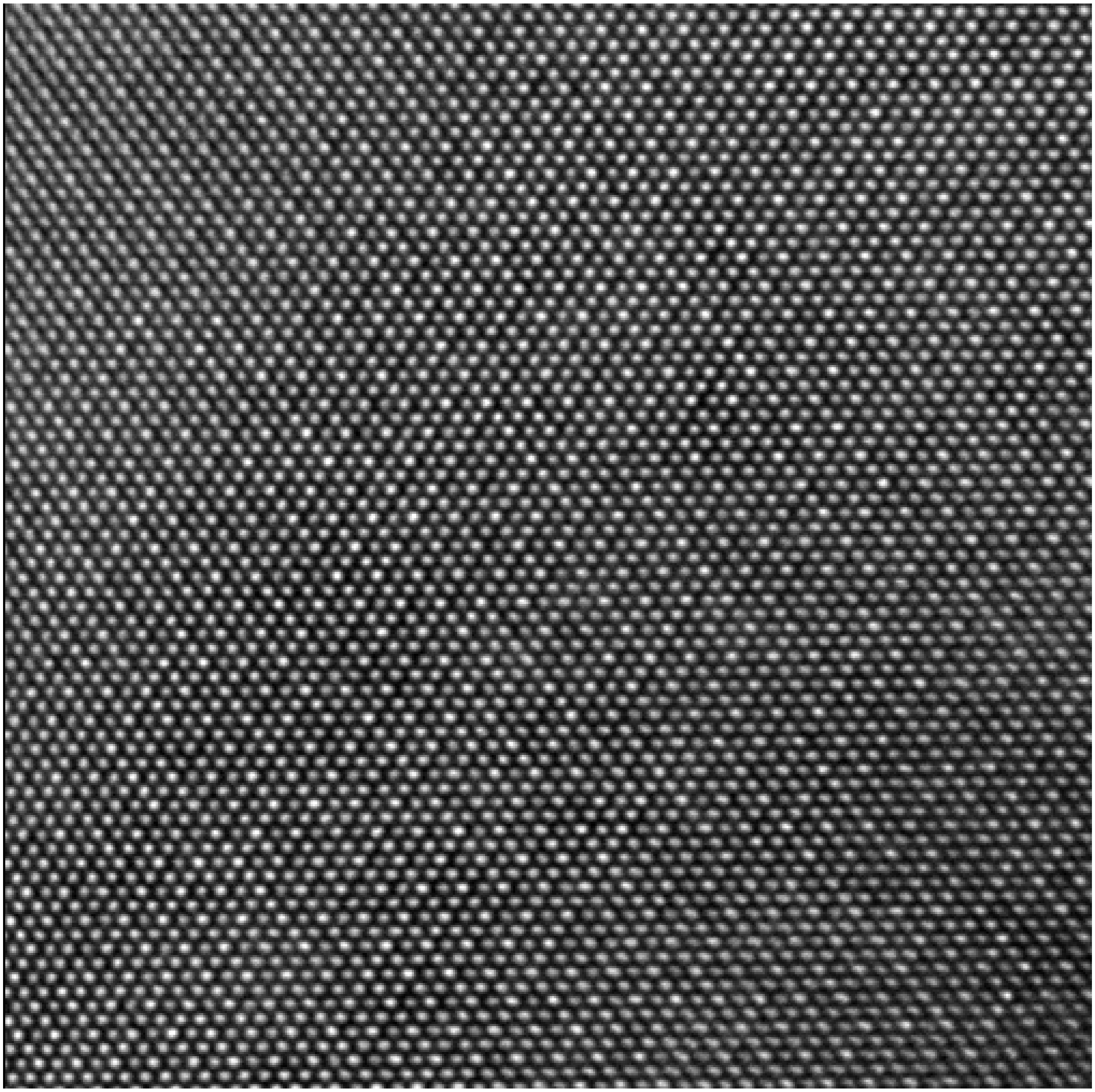

Supplementary Figure 44| Representative zoomed out $Pr_2Zr_2O_7$ [110] HAADF STEM image.

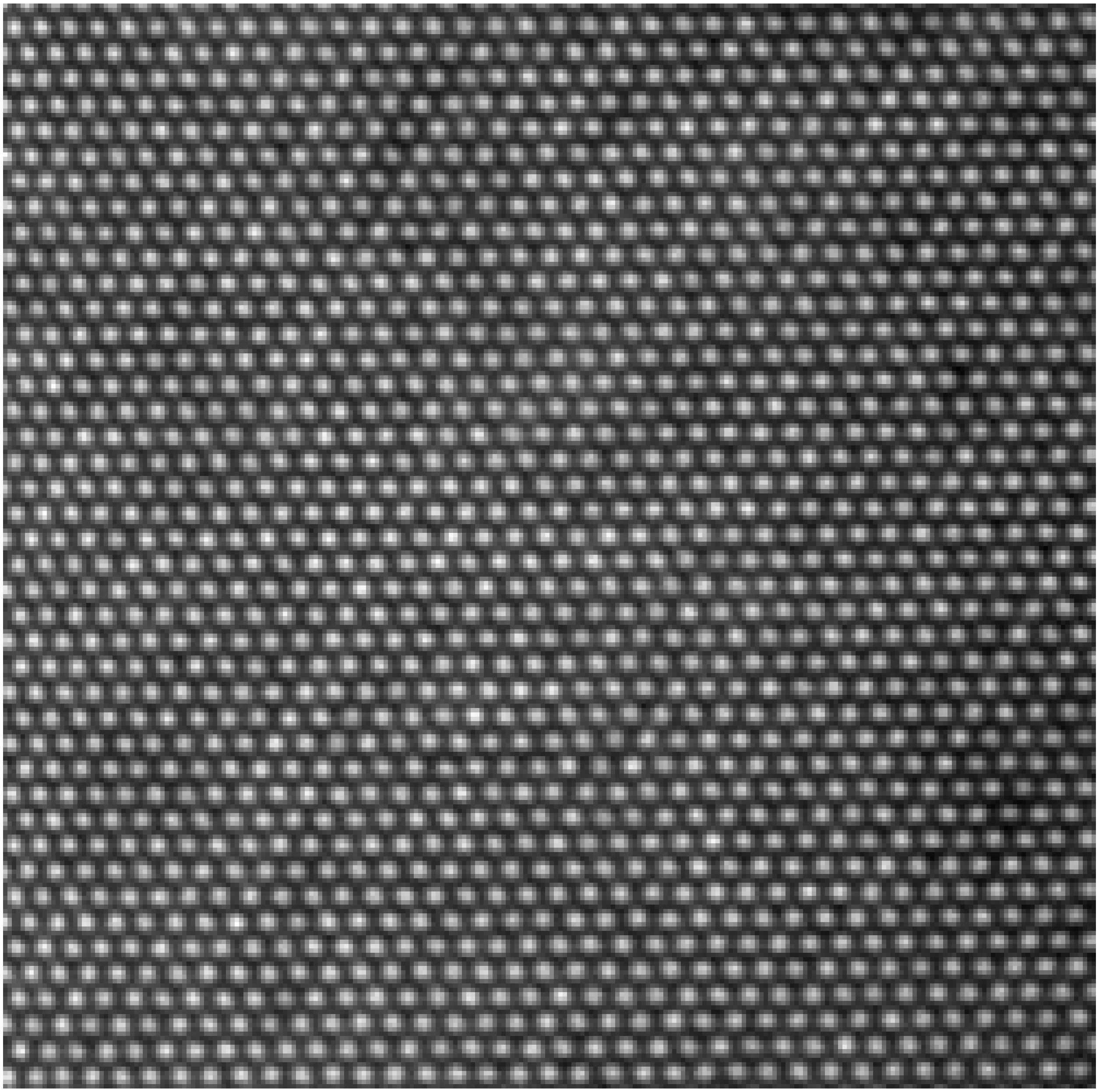

Supplementary Figure 45| Representative zoomed out $Pr_2Zr_2O_7$ [111] HAADF STEM image.

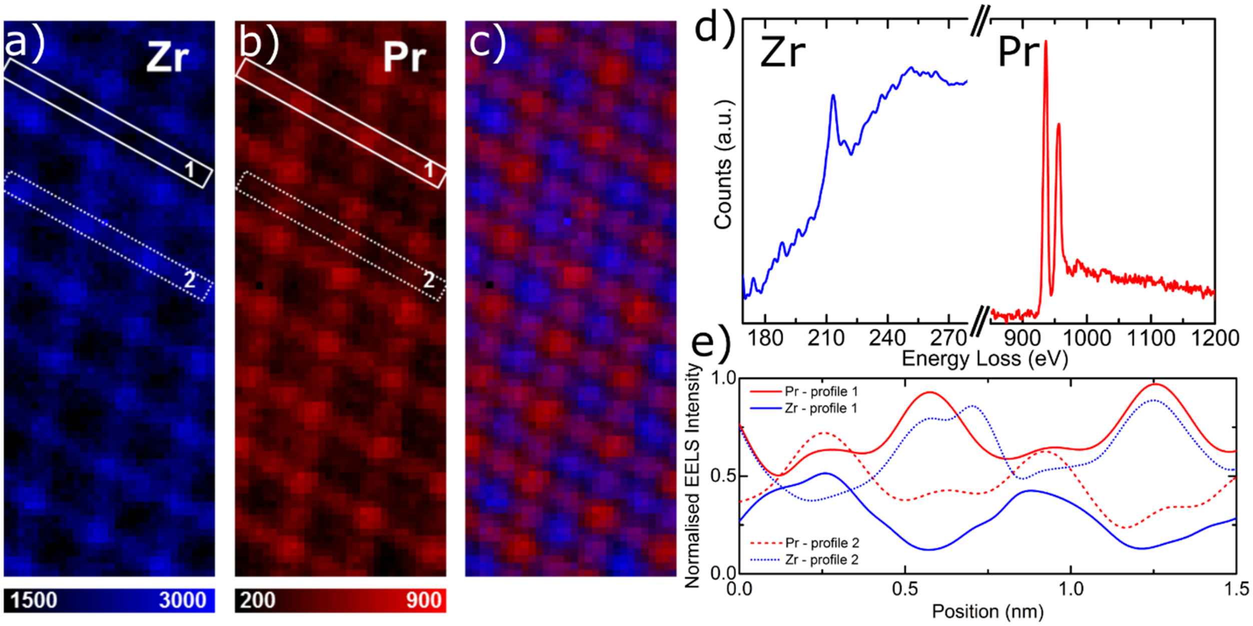

Supplementary Figure 46| Representative Electron Energy Loss Spectrum dataset. Images processed as discussed above. The a) Zr and b) Pr $M_{4,5}$ maps are shown, along with the c) composite color map. While the images are visualized after normalization to unity, the corresponding total counts are indicated on the color scales below. d) Zr and Pr $M_{4,5}$ spectra (averaged over the entire spectrum image and deconvolved for removal of plural scattering effects) are presented. e) Finally, lines profiles taken across the structure as indicated show the uniformity of the EELS intensity distribution (within the measurement error).

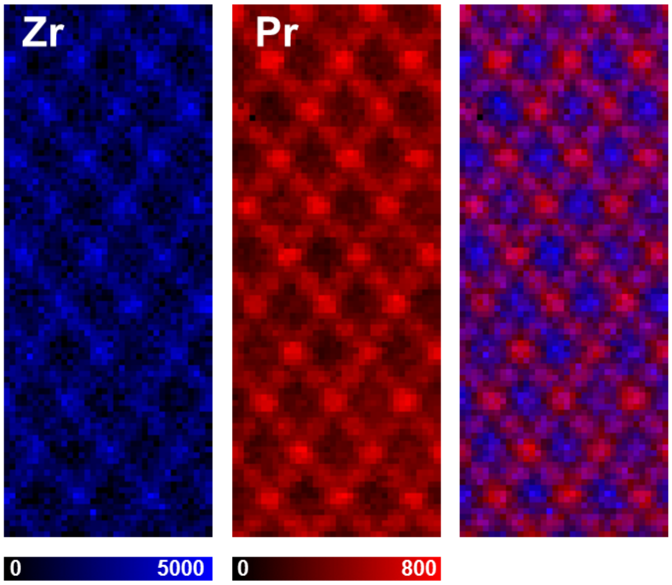

Supplementary Figure 47| Zr and Pr chemical maps. Images are processed as discussed in main text, corresponding to the data used in Figure 5 of the main text, presented here for completeness.

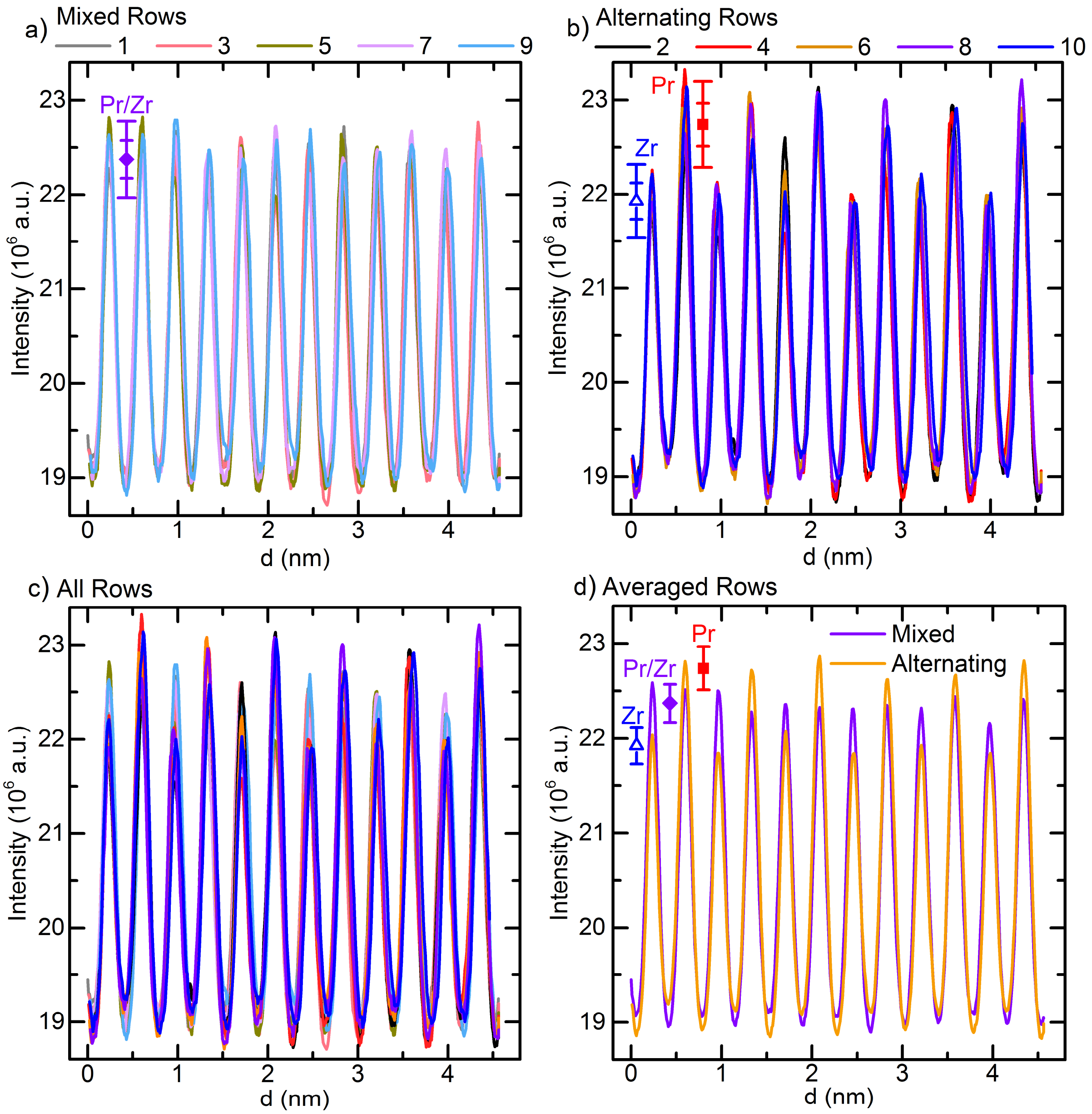

Supplementary Figure 48 | Intensity profiles for a $Pr_2Zr_2O_7$ crystal oriented in the [110] direction. Profiles are for a) individual mixed column rows, b) individual alternating rows which show alternating columns of Pr and Zr, c) all individual rows, and d) averages of mixed (purple) and alternating (orange) rows. The purple diamond represents average mixed column intensity, the red square represents average Pr column intensity, and the blue triangle represents average Zr column intensity, with two standard deviations shown in a) and b).

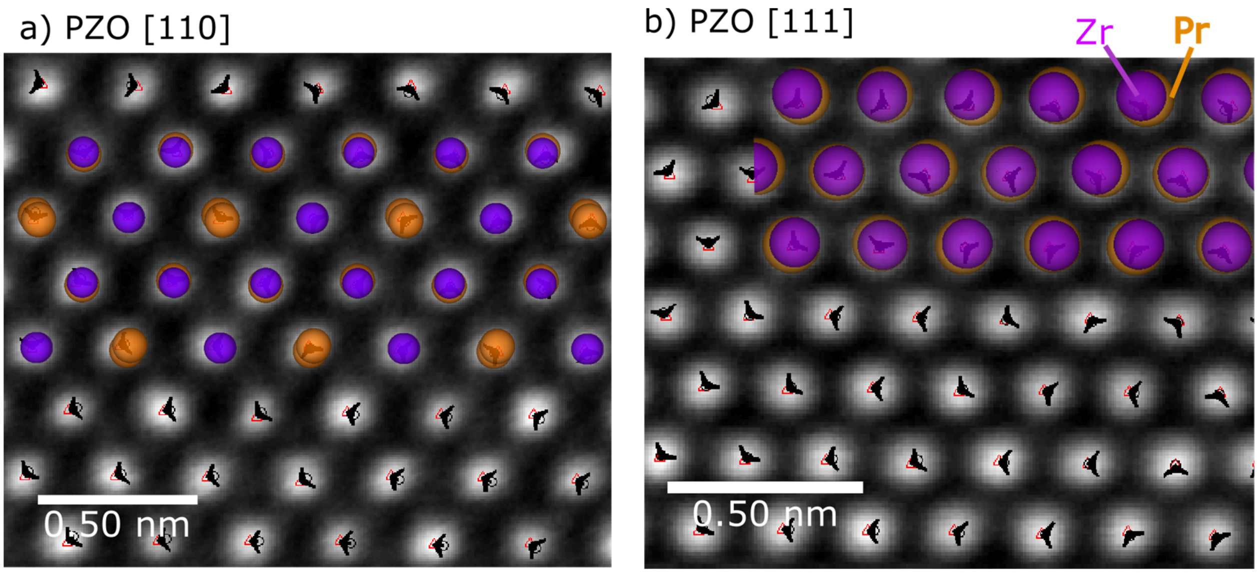

Supplementary Figure 49| High Angle Annular Dark Field Scanning Tunneling Microscopy images of $Pr_2Zr_2O_7$. Orientations are in the a) [110] and b) [111] directions, with the $P4_32_12$ structure from NPDF refinements overlaid. Pr are shown in orange and Zr in purple. Arrowheads represent a displacement vector from the ideal $Fd\overline{3}m$ structure (black circles) to experimental (red triangles) positions.

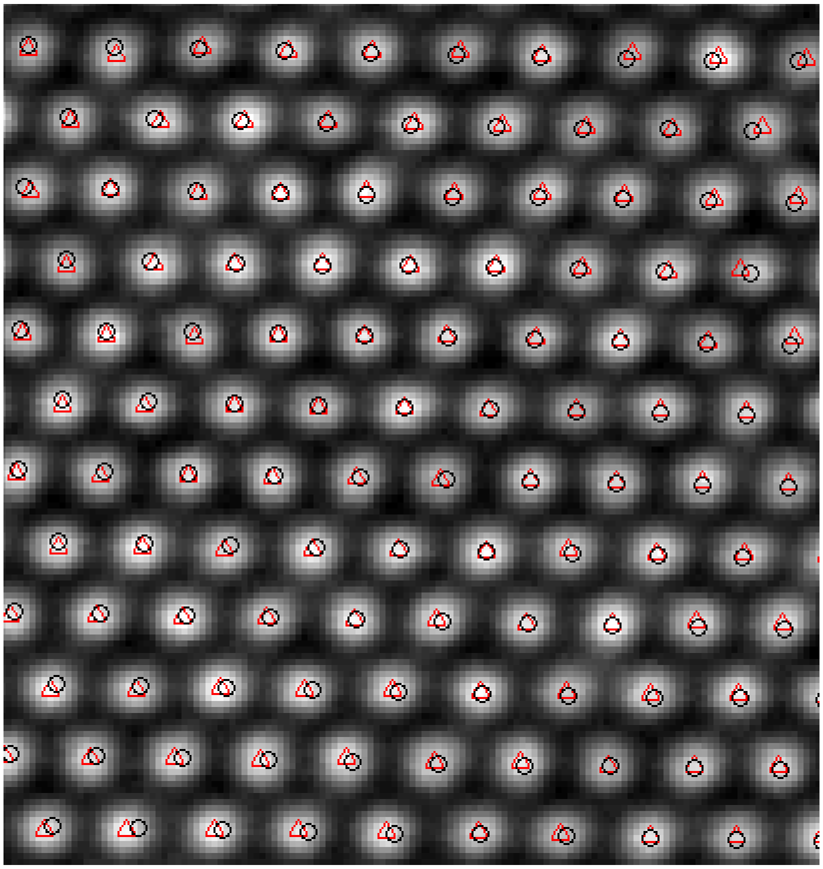

Supplementary Figure 50 | Zoomed out $Pr_2Zr_2O_7$ [111] High Angle Annular Dark Field Scanning Tunneling Microscopy image used for additional data for the histogram in Figure 6b. Red triangles represent experimental atomic column positions with black circles representing ideal $Fd\bar{3}m$ structure.

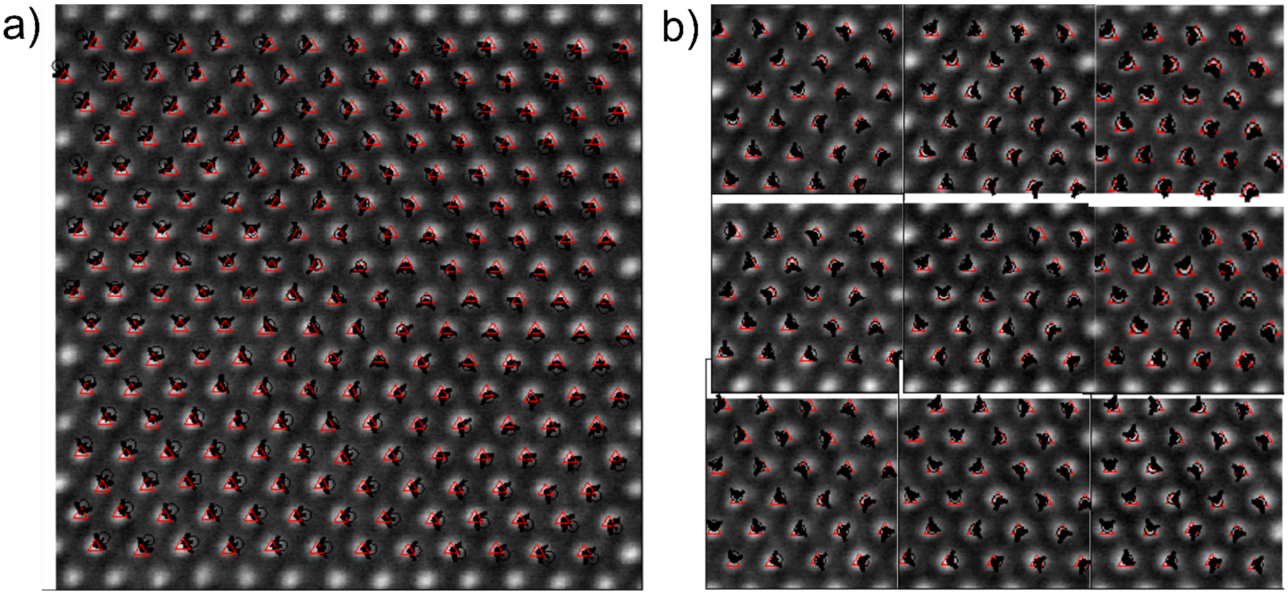

Supplementary Figure 51| High Angle Annular Dark Field Scanning Tunneling Microscopy image for $Pr_2Zr_2O_7$ in the [110] direction. a) Fits of ideal $Fd\overline{3}m$ structure (black circles) to experimental positions (red triangles) fits well in the center, but poorly at the edges, especially poor in the upper left and lower right corners. b) Same image split into nine parts fits as well for each portion as for the center in the original image.

	a = *b* (Å)	7.564(3)	V (Å^3)	610.5(7)	
	c (Å)	10.697(4)	R_w (%)	8.757	
	T (K)	298	rχ^2 (%)	5.602	
Atom	Wyck. Pos.	*x*	*y*	*z*	U_{iso} (Å^2)
Pr	8*b*	0.751(3)	0.4996(16)	0.8871(10)	0.0023(10)
Zr	8*b*	0.245(3)	0.496(3)	0.877(2)	0.0060(12)
O'	4*a*	= 0.75	= *x*	= 0	0.0067(12)
O1	8*b*	0.462(3)	0.957(3)	0.750(2)	0.0069(9)
O2	8*b*	0.734(2)	0.243(3)	0.4672(14)	=U_{iso}(O1)
O3	4*a*	0.458(3)	= *x*	= 0	=U_{iso}(O1)
O4	4*a*	0.051(2)	= *x*	= 0	=U_{iso}(O1)

Supplementary Table 1| Crystallographic parameters for $Pr_2Zr_2O_7$ using space group *P*4_32_12(96) obtained from refinement to time of flight neutron pair-distribution data at room temperature. Atoms are restricted by symmetry as 8*b*:(*x*,*y*,*z*) and 4*a*:(*x*,*x*,0). Atomic displacement parameters for O (except O') were constrained with each other. O' was fixed at its nominal position and all occupancies were fixed at unity, both due to systematic refinement tests. Errors represent statistical uncertainties. See Supplemental Data 1 for CIF.

	a = *b* (Å)	7.0970(8)	*V* (Å^3)	505.53(10)	
	c (Å)	10.0367(12)	R_w (%)	8.677	
	T (K)	298	rχ^2 (%)	24.796	
Atom	Wyck. Pos.	*x*	*y*	*z*	U_{iso} (Å^2)
Yb	8*b*	0.7551(9)	0.4951(12)	0.8789(8)	0.0058(6)
Ti	8*b*	0.250(2)	0.500(4)	0.884(3)	0.0063(10)
O'	4*a*	= 0.75	= x	= 0	0.014(4)
O1	8*b*	0.456(5)	0.949(7)	0.749(4)	0.0078(10)
O2	8*b*	0.800(3)	0.306(5)	0.457(3)	=U_{iso}(O1)
O3	4*a*	0.5420(14)	= *x*	= 0	=U_{iso}(O1)
O4	4*a*	0.0461(6)	= *x*	= 0	=U_{iso}(O1)

Supplementary Table 2| Crystallographic parameters for $Yb_2Ti_2O_7$ using space group *P*4_32_12(96) obtained from refinement to synchrotron X-ray pair-distribution data at room temperature. Atoms are restricted by symmetry as 8*b*:(*x*,*y*,*z*) and 4*a*:(*x*,*x*,0). Atomic displacement parameters for O (except O') were constrained with each other. O' was fixed at its nominal position and all occupancies were fixed at unity, both due to systematic refinement tests. Errors represent statistical uncertainties. See Supplemental Data 2 for CIF.

References

1. Subramanian, M. A., Aravamudan, G. & Subba Rao, G. V. Oxide pyrochlores — A review. *Prog. Solid State Chem.* **15,** 55–143 (1983).

2. Shamblin, J. *et al.* Probing disorder in isometric pyrochlore and related complex oxides. *Nat. Mater.* **15,** 507–512 (2016).

3. Tabira, Y., Withers, R. L., Minervini, L. & Grimes, R. W. Systematic structural change in selected rare earth oxide pyrochlores as determined by wide-angle CBED and a comparison with the results of atomistic computer simulation. *J. Solid State Chem.* **153,** 16–25 (2000).

4. Wilde, P. J. & Catlow, C. R. A. Defects and diffusion in pyrochlore structured oxides. *Solid State Ionics* **112,** 173–183 (1998).

5. Moriga, T. *et al.* Crystal structure analyses of the pyrochlore and fluorite-type $Zr_2Gd_2O_7$ and anti-phase domain structure. *Solid State Ionics* **31,** 319–328 (1989).

6. Clancy, J. P. *et al.* X-ray scattering study of pyrochlore iridates: crystal structure, electronic and magnetic excitations. *Phys. Rev. B* **94,** 024408 (2016).

7. Shoemaker, D. P., Llobet, A., Tachibana, M. & Seshadri, R. Reverse Monte Carlo neutron scattering study of the 'ordered-ice' oxide pyrochlore $Pb_2Ru_2O_{6.5}$. *J. Phys. Condens. Matter* **23,** 315404 (2011).

8. Ueda, K. *et al.* Magnetic Field-Induced Insulator-Semimetal Transition in a Pyrochlore $Nd_2Ir_2O_7$. *Phys. Rev. Lett.* **115,** 056402 (2015).

9. Thygesen, P. M. M. *et al.* Orbital Dimer Model for the Spin-Glass State in $Y_2Mo_2O_7$. *Phys. Rev. Lett.* **118,** 067201 (2017).

10. McQueen, T. M. *et al.* Frustrated ferroelectricity in niobate pyrochlores. *J. Phys. Condens. Matter* **20,** 235210 (2008).

11. Booth, C. *et al.* Local lattice disorder in the geometrically frustrated spin-glass pyrochlore $Y_2Mo_2O_7$. *Phys. Rev. B* **62,** R755–R758 (2000).

12. Hatch, D. M. & Ghose, S. The α-β phase transition in cristobalite, SiO_2. *Phys. Chem. Miner.* **17,** 554–562 (1991).

13. Goodwin, A. L., Withers, R. L. & Nguyen, H.-B. Real-space refinement of single-crystal electron diffuse scattering and its application to $Bi_2Ru_2O_{7-\delta}$. *J. Phys. Condens. Matter* **19,** 335216 (2007).

14. Shoemaker, D. P. *et al.* Atomic displacements in the charge ice pyrochlore $Bi_2Ti_2O_6O'$ studied by neutron total scattering. *Phys. Rev. B* **81,** 144113 (2010).

15. Shoemaker, D. P., Seshadri, R., Tachibana, M. & Hector, A. L. Incoherent Bi off-centering in $Bi_2Ti_2O_6O'$. *Phys. Rev. B* **84,** 064117 (2011).

16. Tabira, Y., Withers, R., Thompson, J. & Schmid, S. Structured Diffuse Scattering as an Indicator of Inherent Cristobalite-like Displacive Flexibility in the Rare Earth Zirconate Pyrochlore $La_\delta Zr_{1-\delta}O_{2-\delta/2}$, 0.49<δ<0.51. *J. Solid State Chem.* **142,** 393–399 (1999).

17. Tabira, Y., Withers, R. L., Yamada, T. & Ishizawa, N. Annular dynamical disorder of the rare earth

ions in a $La_2Zr_2O_7$ pyrochlore via single crystal synchrotron X-ray diffraction. *Zeitschrift fur Krist.* **216,** 92–98 (2001).

18. Greedan, J. E. *et al.* Local and average structures of the spin-glass pyrochlore Y2Mo2O7 from neutron diffraction and neutron pair distribution function analysis. *Phys. Rev. B* **79,** 014427 (2009).
