# Supplementary material for: Universal geometric frustration in pyrochlores
Source: Nat Commun. 2018 Jul 5;9:2619. doi: 10.1038/s41467-018-05033-7 (PMC6033937; doi:10.1038/s41467-018-05033-7)
Supplement: Supplementary file 3 — Description of Additional Supplementary Files [file 41467_2018_5033_MOESM3_ESM.pdf]

Description of Additional Supplementary Files

File Name: Supplementary Data 1
Description: CIF file for $Pr_2Zr_2O_7$ using space group $P4_32_12$.

File Name: Supplementary Data 2
Description: CIF file for $Yb_2Ti_2O_7$ using space group $P4_32_12$.
